# A chemical approach for global protein knockdown from mice to non-human primates

**DOI:** 10.1038/s41421-018-0079-1

**Published:** 2019-02-05

**Authors:** Xiuyun Sun, Jun Wang, Xia Yao, Wen Zheng, Yang Mao, Tianlong Lan, Liguo Wang, Yonghui Sun, Xinyi Zhang, Qiuye Zhao, Jianguo Zhao, Rui-Ping Xiao, Xiuqin Zhang, Guangju Ji, Yu Rao

**Affiliations:** 1 Ministry of Education (MOE) Key Laboratory of Protein Sciences, School of Pharmaceutical Sciences, Tsinghua University, Beijing, 100084 China; 2 MOE Key Laboratory of Bioorganic Phosphorus Chemistry and Chemical Biology, Beijing Advanced Innovation Center for Structural Biology, Tsinghua University, Beijing, 100084 China; 3 Tsinghua-Peking Center for Life Sciences, Haidian District, Beijing, 100084 China; 4 Institute of Biophysics, Chinese Academy of Sciences, Beijing, 100101 China; 5 University of the Chinese Academy of Sciences, Beijing, 100049 China; 6 Institute of Molecular Medicine, Peking University, Beijing, 100871 China; 7 Beijing Key Laboratory of Cardiometabolic Molecular Medicine, Peking University, Beijing, 100871 China; 8 State Key Laboratory of Stem Cell and Reproductive Biology, Institute of Zoology, Chinese Academy of Sciences, Beijing, 100101 China; 9 State Key Laboratory of Membrane Biology, Institute of Molecular Medicine, Peking University, Beijing, 100871 China; 10 Peking-Tsinghua Center for Life Sciences, Beijing, 100871 China

**Keywords:** Proteolysis, Biological techniques

## Abstract

Although conventional genetic modification approaches for protein knockdown work very successfully due to the increasing use of CRISPR/Cas9, effective techniques for achieving protein depletion in adult animals, especially in large animals such as non-human primates, are lacking. Here, we report a chemical approach based on PROTACs technology that efficiently and quickly knocks down FKBP12 (12-kDa FK506-binding) protein globally in vivo. Both intraperitoneal and oral administration led to rapid, robust, and reversible FKBP12 degradation in mice. The efficiency and practicality of this method were successfully demonstrated in both large and small animals (mice, rats, Bama pigs, and rhesus monkeys). Furthermore, we showed this approach can also be applied to effectively knockdown other target proteins such as Bruton's tyrosine kinase (BTK). This chemical protein knockdown strategy provides a powerful research tool for gene function studies in animals, particularly in large animals, for which gene-targeted knockout strategies may remain unfeasible.

## Introduction

Animal models with protein depletion represent a powerful strategy to investigate the functional consequence of the loss of a target gene^1^. Traditionally, loss-of-function studies of genes have mainly been achieved through genetic modifications by RNA interference^2^, transcription activator-like effector nucleases^3,4^, recombination-based gene knock-out, clustered regularly interspaced short palindromic repeats (CRISPR)-Cas9 system^5,6^, and other genome editing strategies. However, it remains challenging to apply these strategies in large animals, particularly in non-human primates, which have recently gained broad attention in both fundamental research and the drug-discovery industries. Besides, these approaches have failed at a certain degree to control acute and reversible changes of gene function^7^. Furthermore, the complications of potential genetic compensation and/or spontaneous mutations arising in gene-knockout models may lead to misinterpretations^8–10^. In addition, deletions of many genes result in embryonic lethality of animals, which hampers the related scientific research^11^.

**Fig. 1:**
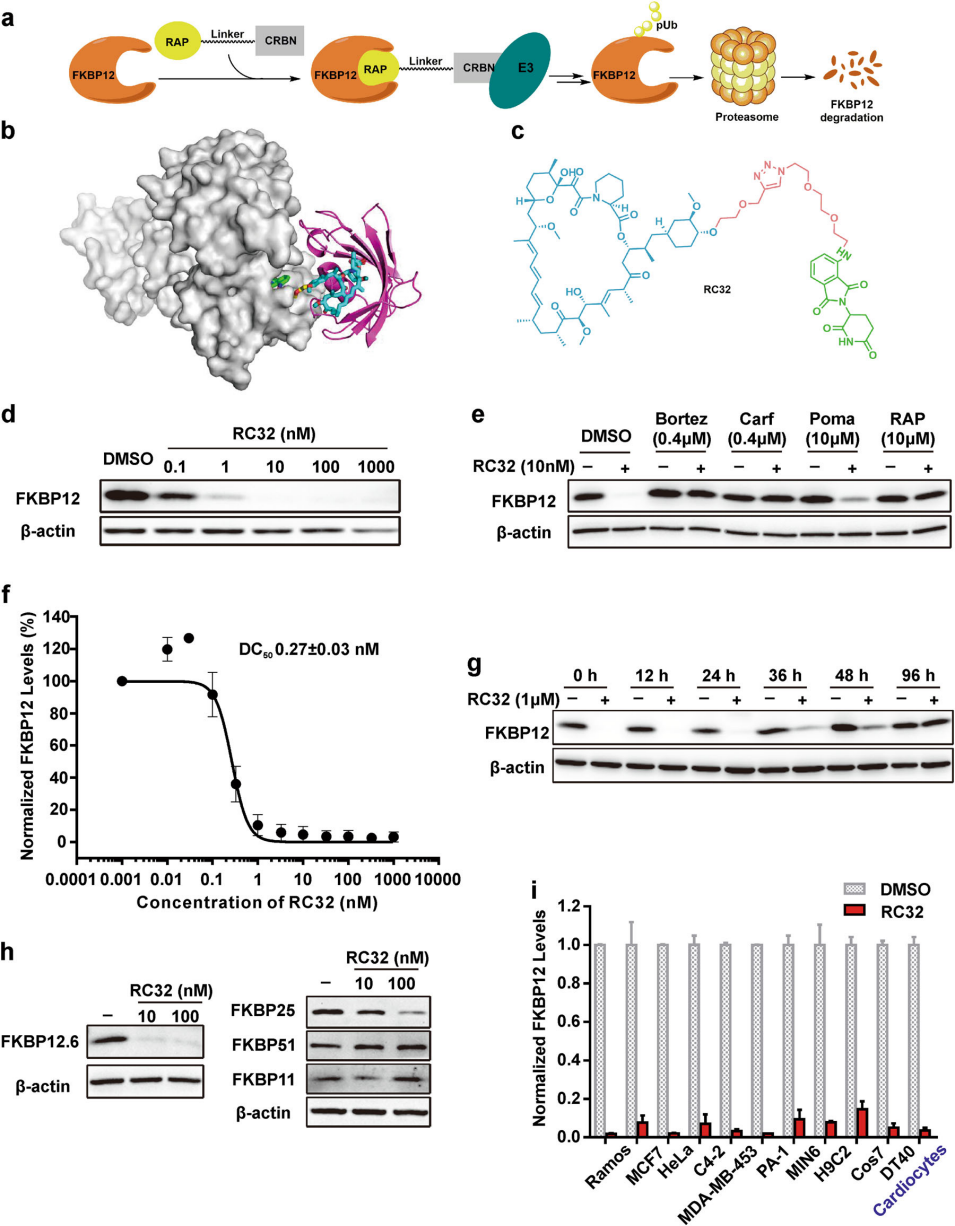
RC32-induced degradation of FKBP12 in cell cultures. **a** Mechanism of action of PROTAC. **b** Docking mode of RC32 binding to FKBP12 and recruiting CRBN. Moiety in red, linker; moiety in blue, rapamycin; moiety in green, pomalimide. **c** Chemical structure of RC32. **d** Immunoblots showing degradation of FKBP12 in Jurkat cells treated with RC32 at the indicated concentrations for 12 h. ß-Actin served as a loading control. **e** Immunoblots for FKBP12 protein. Jurkat cells were first treated for 3 h with bortezomib (Bortez), carfilzomib (Carf), rapamycin (RAP), or pomalimide (Poma) followed by treatment with RC32 (10 nM) for 2 h. ß-Actin served as a loading control. **f** DC_50_ with 12 h of RC32 treatment in Jurkat cells. **g** Recovery of FKBP12 level after washout of RC32. After treatment for 12 h with 1 μM RC32, the Jurkat cells were washed with fresh culture medium and further cultured in fresh medium for the indicated times. **h** Selectivity of RC32 against FKBPs in Jurkat cells. **i** Degradation efficiency of RC32 in different cell lines (100 nM) and primary cardiac myocytes (1 µM) treated for 12 h. Data in (**f**) and (**i**) are presented as mean ± SEM of 3 independent experiments

Proteolysis-targeting chimeras (PROTACs)^12–14^ contain a specific ligand for a target protein of interest that is connected via a linker to a ligand for an E3 ubiquitin ligase. PROTACs represent a chemical knockdown strategy that operates through the formation of a trimeric complex that allows the ubiquitination and subsequent degradation of the target protein via the proteasome (Fig. 1a). Following the development of the first small molecule-based PROTAC which was reported by Crews group, a growing number of interested proteins have been successfully degraded through PROTACs approach. At present, PROTACs are mainly applied in the discovery of new anti-cancer agents due to their unique advantages over classic inhibitors^15–17^. To our knowledge, this novel strategy has not been used to achieve global protein knockdown in large animal models in vivo. A model species that is closely related to humans, the rhesus monkey, is a unique model for studying various diseases due to its human-like genome, the controllability of environmental factors, and the feasibility of monitoring the metabolic phenotypes in real time. However, it is unknown whether PROTACs work in non-human primates.

Here, we demonstrate that PROTAC technology, a convenient, fast, and reversible chemical approach, can degrade proteins globally and quickly (24–72 h) in living animals, specifically in pigs and rhesus monkeys. After withdrawal of PROTAC, the protein level recovered, suggesting that this method is cost-effective and time-efficient for use in self-controlled animal studies. Furthermore, we investigated the importance of FKBP12 in the maintenance of cardiac functions using FKBP12 knockdown generated by PROTACs in mice and rhesus monkeys.

## Results

### Design and characterization of FKBP12-targeting PROTACs

In this study, we chose FKBP12 as the initial target protein. The reasons are as follows.

First, FKBP12 protein is widely expressed in mammals. Through binding the Ca^2+^-release channel (ryanodine receptor), FKBP12 regulates Ca^2+^ signaling to carry out important functions^18^, particularly in the heart^19–22^. Second, because FKBP12 is a highly conserved protein, this chemical knockdown strategy is promising for creating large animal models (e.g., pigs and non-human primates) with targeted protein knockdown. Third, the global knockdown of FKBP12 via prevalent approaches is embryonic lethal due to severe developmental heart defects such as hypertrabeculation, ventricular non-compaction, and ventricular septal defects^23^. To date, the role of FKBP12 in cardiac function and disease development in adults remains elusive. Moreover, it has been demonstrated that rapamycin^24,25^ is a potent and specific ligand of FKBP12 that modulates the mTOR (mammalian target of rapamycin) signaling with a high binding affinity (Kd 0.2 nM)^26^. Thus, we designed FKBP12-targeting chimeras by linking rapamycin (an FKBP12-specific ligand) and pomalidomide [a CRBN (E3 protein ligase complex)-specific ligand] through polyethylene glycol linkers (Fig. 1b). When the heterobifunctional molecule binds to FKBP12 via the rapamycin moiety, the ubiquitination of FKBP12 occurs via an E3 ligase (CRBN) that is recruited by the pomalidomide component and results in the subsequent degradation of FKBP12 via the proteasome. So, we synthesized a series of PROTACs with E3 ligase ligands and rapamycin and assayed their potential to degrade FKBP12 in Jurkat cells.

A compound named RC32 (Fig. 1c) with conjugation of rapamycin and pomalidomide demonstrated the most potent ability for FKBP12 degradation with a concentration that resulted in 50% protein degradation (DC_50_) of ~0.3 nM after only 12 h of treatment (Fig. 1d, f, Supplementary Fig. S2).

To determine whether RC32-induced ubiquitination leads to FKBP12 degradation via the ubiquitin–proteasome system (Fig. 1e), cells were treated with the proteasome inhibitors bortezomib or carfilzomib prior to RC32 application. Indeed, inhibition of proteasome completely blocked the RC32-induced degradation of FKBP12, indicating that this degradation depends on the ubiquitin–proteasome system. Addition of the FKBP12 binder rapamycin or the CRBN binder pomalidomide effectively rescued the degradation of FKBP12 by RC32, further confirming that this degradation requires the binding of RC32 to FKBP12 and CRBN. Notably, while degrading FKBP12.6, RC32 induced no evident degradation of FKBP51 and FKBP11 in Jurkat cells (Fig. 1h, Supplementary Fig. S12). Meanwhile, the degradation of FKBP25 could be controlled when used with proper RC32 dose (Supplementary Fig. S13). Furthermore, RC32 had no influence on the phosphorylation of S6K and S6 (Supplementary Figs. S3, S11), which could be a benefit in delineating the mTOR-independent function of FKBP12^27,28^. When RC32 was washed out, the FKBP12 protein level fully recovered in 96 h (Fig. 1g). To further assess the efficiency of RC32, different cell lines from different species and primary cells were used for detection (Fig. 1i). FKBP12 was efficiently degraded by RC32 in cells from humans, rats, mice, and chickens in a highly consistent pattern. Importantly, RC32 demonstrated high degradation efficiency in primary cardiac myocytes, which suggested its potential for degrading FKBP12 in vivo.

### Rapid and potent degradation of FKBP12 in mice and rats by RC32

After confirming the high degradation potency in cell lines and primary cells, we endeavored to establish RC32-mediated protein knockdown in mouse, rat, pig, and non-human primate animal models. These models are invaluable tools for studying human diseases. While mice and rats have been used extensively in scientific research, it is much more difficult to construct protein knockdown models in pigs and non-human primates. Pigs are particularly attractive for xeno-transplantation in biomedical research^29^. Although non-human primates are more relevant for studying human disease and developing therapeutic strategies^30^, genetic modifications in monkeys remain expensive, time-consuming, and technically challenging. Their limitations in large animal models have markedly hindered their use in biomedical research. Therefore, we attempted to apply this chemical approach to construct representative protein-knockdown animal models and study the functions of the target protein.

**Fig. 2:**
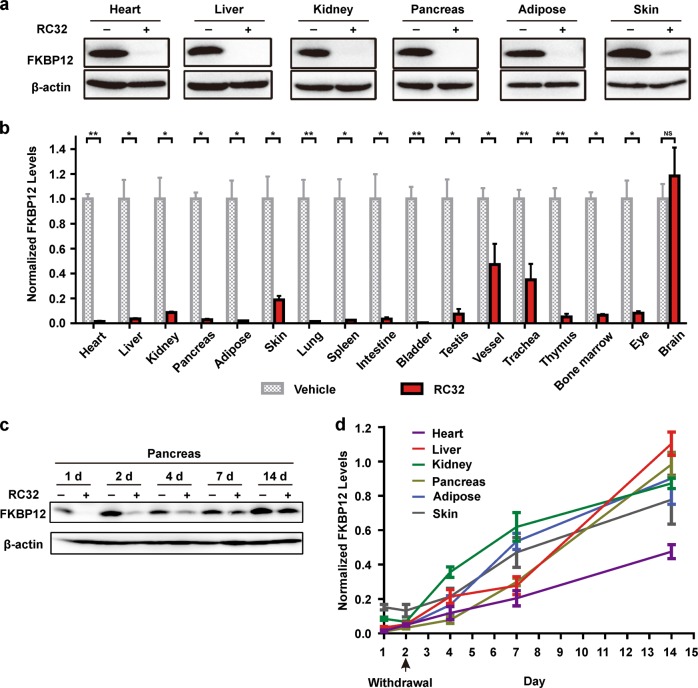
Global knockdown of FKBP12 in mice by i.p. RC32 and recovery of FKBP12 protein levels. **a** Immunoblotting for FKBP12 in various organs/tissues of mice given RC32 (30 mg/kg, i.p., twice a day for 1 day). **b** FKBP12 protein levels in different organs/tissues of mice (RC32 group, *n* = 6; vehicle group, *n* = 4). **c** Immunoblots showing recovery of the FKBP12 level in the pancreas after withdrawal of RC32 at day 2. **d** Recovery of the FKBP12 level in representative organs/tissues after withdrawal of RC32 (*n* = 6). Data in (**b**) and (**d**) are presented as mean ± SEM and assessed with the two-tailed unpaired Student’s *t*-test (*p < 0.05, **p < 0.01; n.s., not significant)

**Fig. 3:**
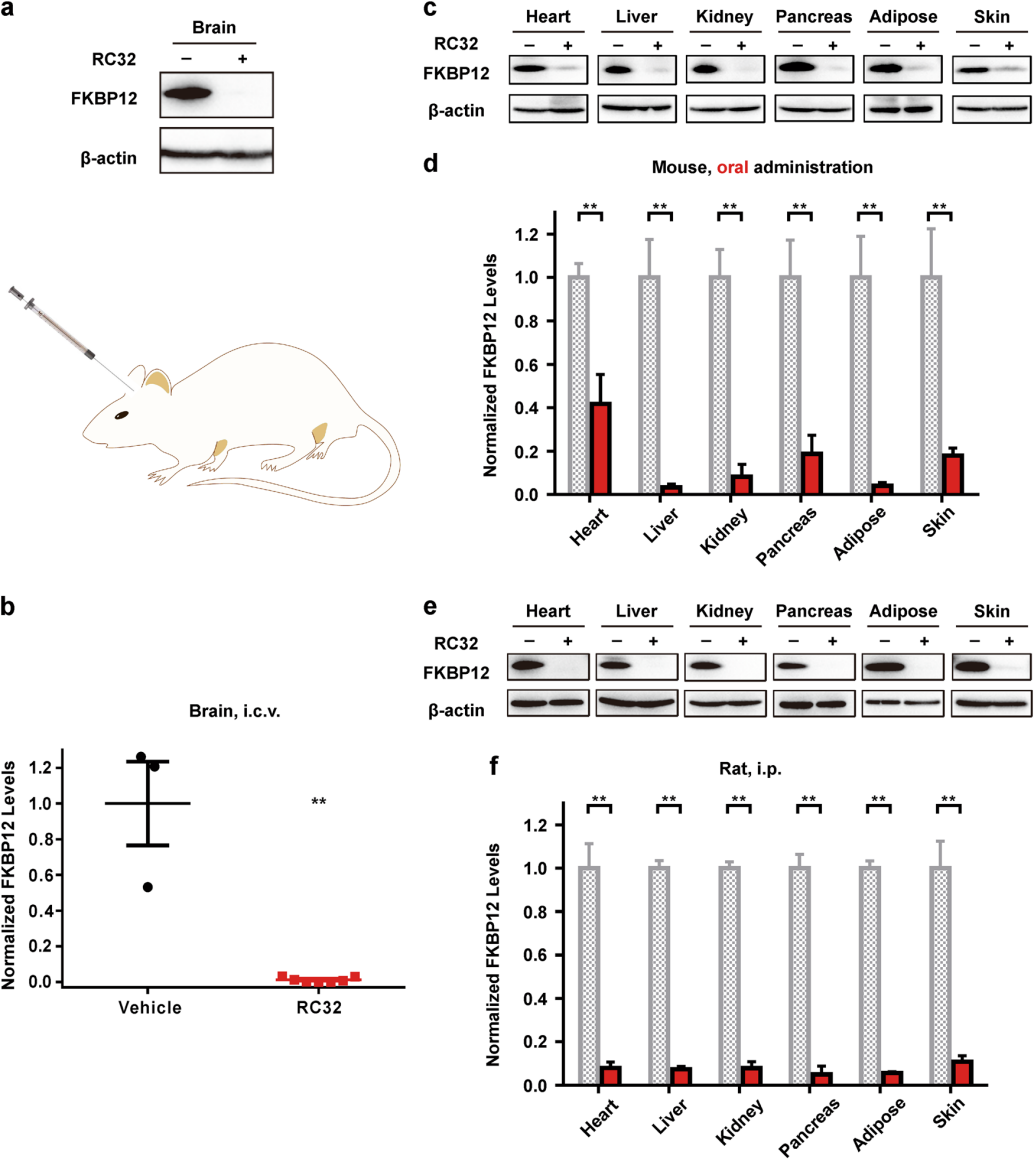
FKBP12 protein knockdown by RC32 after oral administration and i.c.v. in mice and i.p. in rats. **a** Upper panel, immunoblot of FKBP12 degradation in the brain of mice with a single i.c.v. injection (0.2 mg in 2 µL). Lower panel, schematic of i.c.v. injection of RC32. **b** Statistical analysis of the FKBP12 degradation as in (**a**) (RC32 group, *n* = 6; vehicle group, *n* = 3). **c** Immunoblots of FKBP12 in tissues/organs from mice given RC32 orally (60 mg/kg, twice a day for 1 day). **d** Statistical analysis of the FKBP12 degradation as in (**c**) (*n* = 3). **e** Immunoblots of FKBP12 degradation in rats after 1 day of RC32 administration (20 mg/kg, i.p., twice per day). **f** Statistical analysis of the FKBP12 degradation as in (**e**) (*n* = 5). Data in (**b**), (**d**), and (**f**) are presented as mean ± SEM and assessed with the two-tailed unpaired Student’s *t*-test (*p < 0.05, **p < 0.01)

We first investigated its effects in mice. Surprisingly, the FKBP12 protein in most of the organs of treated mice, except for the brain, was undetectable after only 1 day of treatment (intraperitoneal (i.p.) injection, 30 mg/kg, twice a day) with RC32 (Fig. 2a, b). Interestingly, a significant degradation of FKBP12 also occurred in the eyes. In contrast, RC32 had no effect on FKBP12 degradation in brain tissues. This might be due to the inability of RC32 to pass the blood–brain barrier. After 1 day treatment (30 mg/kg, twice a day), RC32 was able to degrade FKBP12 for about 1 week. In addition, FKBP12 levels in different organs/tissues recovered after withdrawal of RC32 (Fig. 2c, d). Interestingly, the recovery rate of FKBP12 in the heart (Supplementary Fig. S4) was the slowest 13 days after RC32 withdrawal. After withdrawal of the PROTAC probe, the mRNA levels of FKBP12 exhibited an acute and compensatory increase and then quickly returned to and remained at nearly normal levels (Supplementary Fig. S5). In order to degrade FKBP12 protein in the brain, we used intracerebroventricular (i.c.v.) administration. As expected, FKBP12 in tissues from the i.c.v.-treated brain was degraded (Fig. 3a, b), while its levels were not affected in the other organs/tissues examined (Supplementary information, Fig. S6a). FKBP12 is ubiquitously expressed in the nervous systems and is known to regulate the localization and processing of amyloid precursor protein. Our results implicate that localized protein knockdown in the brain may open a new avenue for the treatment of Alzheimer’s disease. FKBP12 was significantly degraded in mice when RC32 was delivered orally (Fig. 3c, d, Supplementary information, Fig. S6b), thus highlighting the clinical potential of the oral administration of PROTACs, which is more convenient than the previously-reported injection-dependent PROTACs. Moreover, a high degradation efficiency was found in Sprague–Dawley rats following only two i.p. doses every 12 h (20 mg/kg; Fig. 3e, f).

### Applications of FKBP12-knockdown mice generated by the PROTAC

**Fig. 4:**
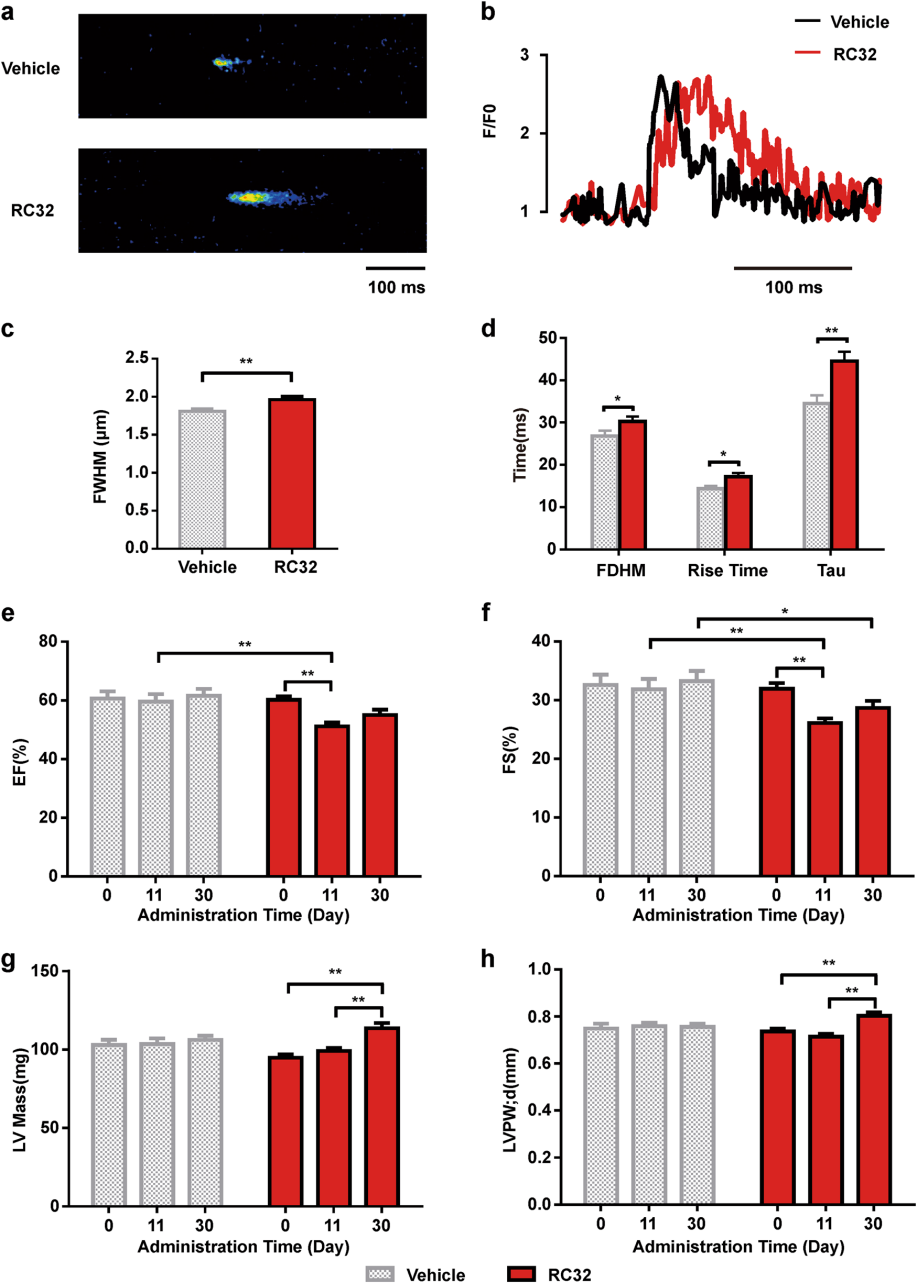
Influence of RC32 administration (30 mg/kg, i.p., twice a day for 2 days and once a day for 28 days) on mouse cardiomyocytes and cardiac function. **a** Representative confocal line-scan images showing Ca^2+^ sparks. **b** Peak of the Ca^2+^ spark. **c** Increased full width at half-maximum (FWHM). **d** Prolonged full-duration at half-maximum (FDHM), rise time, and half-time decay (Tau) (RC32 group, *n* = 39; vehicle group, *n* = 44). **e** Decreased ejection fraction (EF) and **f** fractional shortening (FS) of the mice in the RC32**-**treated group. **g** Increased left ventricular mass (LV mass). **h** Increased left ventricular posterior wall thickness diastolic (LVPW;d) (RC32 group, *n* = 17; vehicle group, *n* = 11). Data in (**c**–**h**) are presented as mean ± SEM. (**c**) and (**d**) were assessed with two-tailed unpaired Student’s *t*-test (*p < 0.05, **p < 0.01) and (**e**–**h**) with two-way ANOVA and the Sidak and Tukey tests (*p < 0.05, **p < 0.01)

FKBP12 is required for heart development, but its physiological importance in cardiac homeostasis remains unclear. We therefore further explored its function(s) in the adult heart using this novel approach in mice. It has been shown that ryanodine receptors are kept in the open state when FKBP12 is depleted, resulting in Ca^2+^ leakage^20^. We then examined the properties of Ca^2+^ sparks in response to the administration of RC32 and found that their duration was greatly prolonged in the RC32-treated group, as shown by confocal microscopic line-scan images of mouse cardiomyocytes treated with vehicle or RC32 (Fig. 4a, b). Compared with controls, the properties of Ca^2+^ sparks were significantly altered in the RC32-treated mouse cardiomyocytes. The full width at half-maximum of the sparks increased from 1.80 ± 0.22 to 1.97 ± 0.26 µm (Fig. 4c). A longer full-duration at half-maximum was found in the PROTAC-treated group (Fig. 4d). The rise-time was prolonged from 14.3 ± 4.67 to 17.2 ± 5.85 ms and the half-time decay from 34.5 ± 12.7 to 44.5 ± 13.5 ms (Fig. 4d). These results suggest that Ca^2+^ leakage is induced in RC32-treated mice. Sustained Ca^2+^ leakage is a trigger of cardiac dysfunction and is the leading cause of cardiac hypertrophy. Next, we examined the cardiac morphology and function in control and RC32-treated mice using echocardiography (Supplementary Fig. S7a). As shown in Fig. 4e, f, the RC32-treated group exhibited a diminished ejection fraction (EF; 14.1%) and a diminished fractional shortening (FS; 18.0%) compared with control hearts, indicating a depression of contractile activity in the ventricular wall of the RC32-treated heart at day 11. When the mice were self-controlled, the EF diminished 14.9% and the FS decreased 18.0% in RC32-treated mice at day 11. In addition, the left ventricular (LV) mass (Fig. 4g, 19.9%), the LV posterior wall thickness, diastolic (LVPW;d) (Fig. 4h, 12.5%), and intraventricular septal width in diastole (IVS;d) (Supplementary Fig. S7b, 5.7%) increased markedly with prolonged RC32 treatment, indicating that RC32-treated mice develop myocardial hypertrophy by day 30. However, the LV mass, LVPW;d, and IVS;d were unchanged in the vehicle group. Due to the development of myocardial hypertrophy, the EF and FS in the RC32-treated group at day 30 were higher than those at day 11. The data characterized by echocardiography illustrated that FKBP12 plays a critical role in the adult heart and that its degradation leads to heart disease. Taken together, these results indicate that knockdown of FKBP12 in mice via the designed PROTAC is a valuable model for studying FKBP12 function, especially in the heart.

### Efficient degradation of FKBP12 in Bama pigs

**Fig. 5:**
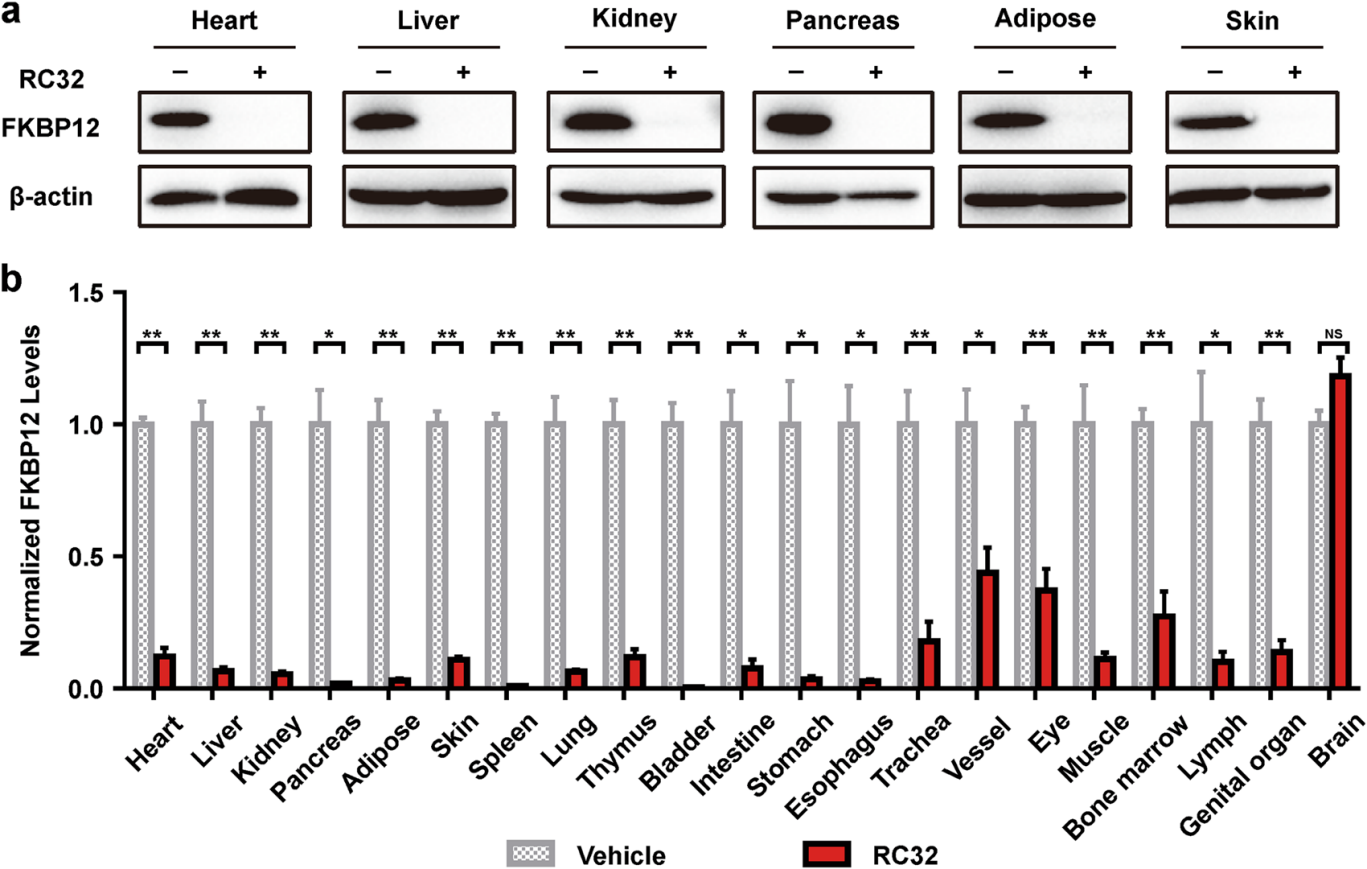
Global knockdown of FKBP12 in Bama pigs by RC32. **a** Efficiency of RC32 (8 mg/kg, i.p., twice a day for 2 days) in pigs. **b** Global knockdown of FKBP12 in different organs/tissues from pigs (RC32 group, *n* = 5; vehicle group, *n* = 3). Data in (**b**) are presented as mean ± SEM and were assessed with the two-tailed unpaired Student’s *t*-test (*p < 0.05, **p < 0.01; n.s., not significant)

Given the above success in mice and rats, we established a protein-knockdown pig model in subsequent experiments. When Bama pigs (20 kg) were given RC32 (8 mg/kg, twice a day) for 2 days, the FKBP12 protein was efficiently degraded in most of the organs examined (Fig. 5a, b, Supplementary Fig. S8). Only residual levels were detectable in the heart, liver, and kidney after RC32 administration. Consistent with our findings in mice, i.p. administration of RC32 had no effect on FKBP12 degradation in the pig brain. Nonetheless, our results demonstrated a remarkable advance in the establishment of protein knockdown models over such a short period using our chemical strategy in large animals. Thus, we believe that the chemical approach inspired by the success of RC32 might generate valuable specific protein knockdown pigs for studies of human disease and xeno-transplantation.

### Validation of the degradation and function of FKBP12 in rhesus monkeys

**Fig. 6:**
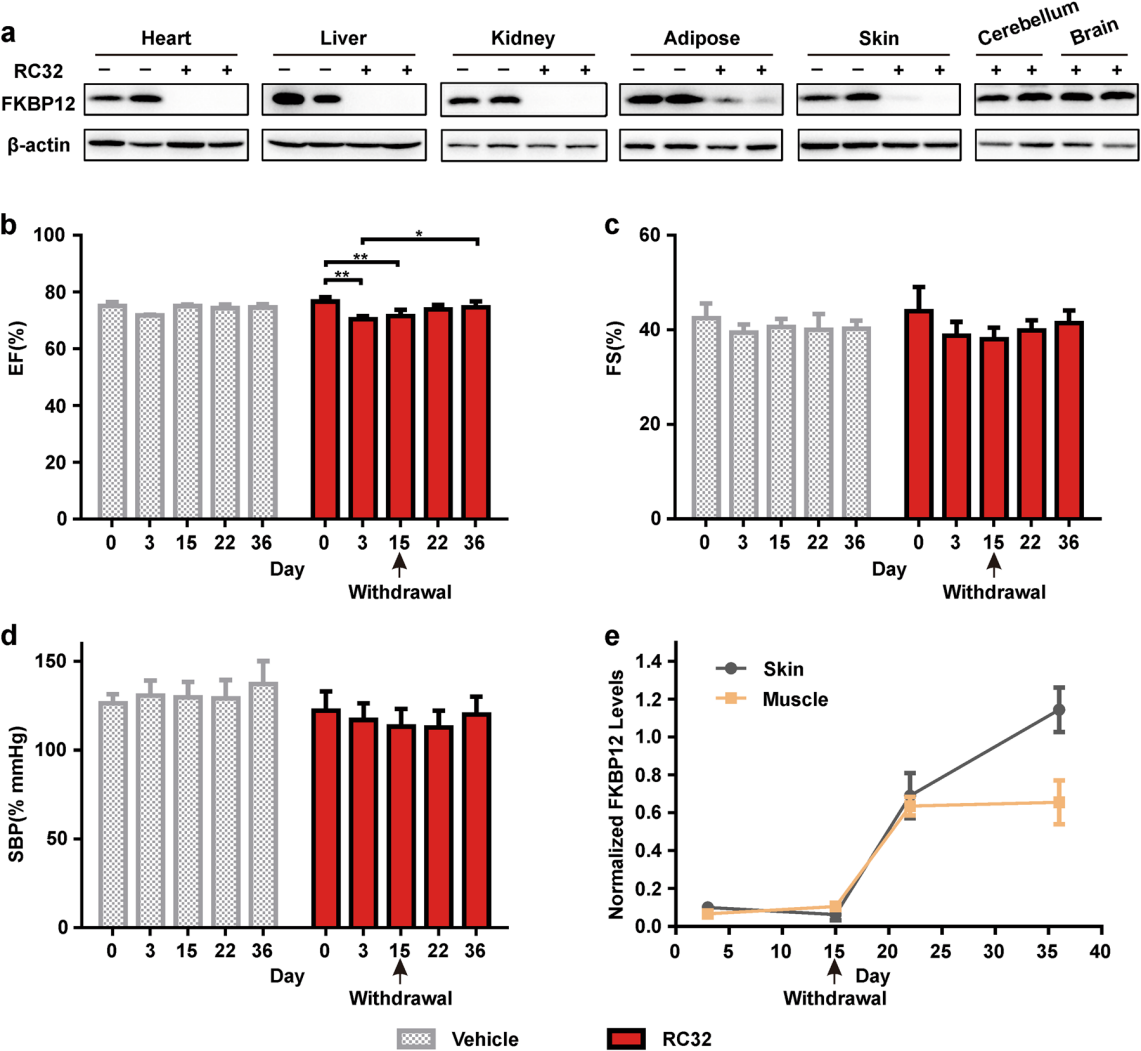
Global knockdown of FKBP12 in rhesus monkeys by RC32 and its functional effects. **a** Efficiency of RC32 knockdown in monkeys (8 mg/kg, i.p., twice a day for 3 days). **b** Decreased EF in the RC32-treated group compared with the vehicle group and recovery after withdrawal of the PROTAC. **c** FS in the RC32-treated group compared with the vehicle group and recovery after withdrawal of the PROTAC. **d** Decreased systolic blood pressure (SBP) in the RC32-treated group compared with the vehicle group and recovery after withdrawal of the PROTAC. **e** FKBP12 protein levels in skin and muscle from the RC32-treated group and recovery of the protein levels after withdrawal of the PROTAC. Data in (**b**–**d**) are presented as mean ± SEM of 3 independent experiments and were assessed with two-way ANOVA with Sidak and Tukey tests (*p < 0.05, **p < 0.01)

Encouraged by the results from the pig model, we extended our studies to rhesus monkeys (Fig. 6). After 3 days of administration (8 mg/kg, i.p., twice a day), FKBP12 in the heart, liver, kidney, spleen, lung, and stomach of monkeys was efficiently degraded by RC32. Little or no FKBP12 was detectable in the adipose tissue, bladder, and intestines (Fig. 6a and Supplementary Fig. S9). RC32 failed to degrade FKBP12 in the brain, consistent with the results from mice and pigs. Moreover, we performed functional heart studies after RC32 administration for 3 and 15 days, as well as at 7 and 21 days after the withdrawal of RC32. Similar to the effect of FKBP12-knockdown on mice, the RC32 treatment reduced EF (6.7% at day 15, Fig. 6b) and FS (13.4% at day 15, Fig. 6c) in the monkeys, in line with the reduced systolic blood pressure in RC32**-**treated monkeys (Fig. 6d). In addition, the heart rate decreased in response to the treatment (Supplementary Fig. S10b). When the administration of RC32 was ceased, the cardiac functions recovered gradually and returned to near normal after 22 days, accompanied by the recovery of FKBP12 protein levels (Fig. 6e, Supplementary Fig. S10a). These results suggest that the chemical protein knockdown method can be used to conduct self-controlled studies. As cardiovascular disease (CVD) is the leading cause of death around the world, the use of this chemical approach to knock down CVD-related proteins provides a novel strategy for studying the pathogenic factors and potential therapies for CVD. More importantly, our studies using the FKBP12 PROTAC probe provide proof-of-principle of this strategy for creating highly efficient and reversible protein knockdown in monkey models.

### Broadening the applicability of the chemical knockdown strategy in BTK

**Fig. 7:**
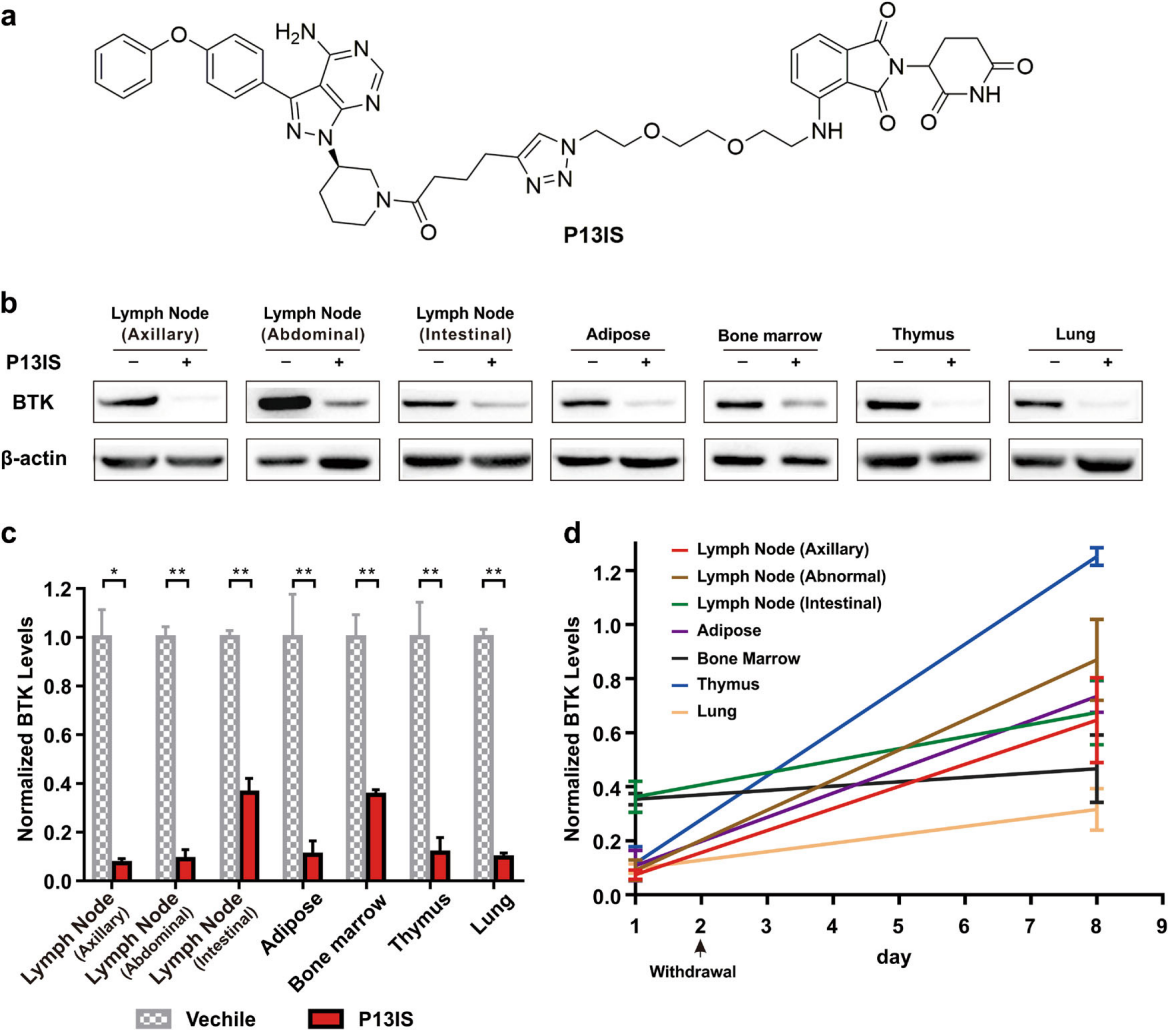
Global knockdown of BTK in mice by P13IS. **a** The chemical structure of P13IS. **b** Efficiency of P13IS (33.3 mg/kg, i.p., three times a day for 11 days) in mice. **c** Global knockdown of BTK in different organs/tissues from mice (*n* = 3). **d** Recovery of the BTK level in representative organs/tissues after withdrawal of P13IS (*n* = 3). Data in (**c**) and (**d**) are presented as mean ± SEM and assessed with the two-tailed unpaired Student’s *t*-test (*p < 0.05, **p < 0.01)

Finally, in order to broaden the applicability of this chemical knockdown approach, Bruton's tyrosine kinase (BTK)-knockdown mice were generated with the further optimized BTK degrader based on our previous studies (Fig. 7a, Supplementary Fig. S14)^31^. After treatment with P13IS (i.p., 33 mg/kg, three times a day) for 11 days, BTK in adipose, thymus, abdominal lymph node, and axillary lymph node was degraded efficiently (Fig. 7b, c). What’s more, a significant degradation of BTK was observed in bone marrow, lung, and intestinal lymph node (Fig. 7b, c). Similar to FKBP12 PROTACs, after withdrawal of P13IS, the BTK level was recovered after 7 days (Fig. 7d).

## Discussion

Taken together, these in vivo protein-degrading results reveal that this approach can be used to establish animal knockdown models at the protein level. This approach can achieve protein knockdown with only days of administration. This technique dramatically reduces the time and cost of establishing an animal model for mechanistic and preclinical studies, especially for larger non-human mammalian models.

In summary, we have developed a chemical approach for the global knockdown of targeted proteins using PROTACs. It is a novel, fast, and effective method for generating protein depletion animal models such as mice, rats, pigs, and rhesus monkeys. Furthermore, this strategy can also achieve conditional protein knockdown in the brain via i.c.v. administration. When the PROTAC probe is administered orally, this approach is also very effective. After withdrawal of the PROTAC, the FKBP12 protein level recovers within a certain period of time. The models are suitable for self-controlled biomedical studies. Mice and rhesus monkeys with FKBP12 knockdown via the chemical strategy exhibited Ca^2+^-leakage and increased Ca^2+^ sparks in cardiomyocytes, and cardiac dysfunctions; thus, these models may be developed for cardiac drug screening. Additionally, this method also can be applied to other targets such as BTK. It may be a challenge to develop PROTAC for those targeted proteins without well-documented specific binders. However, since ligand binding to the target proteins at any site with lower binding affinity is sufficient for PROTAC–target interaction, PROTACs strategy will provide more potentials to achieve efficient protein knockdown. Thus, PROTACs hold the promise to be employed as a complementary approach for currently available tools in a quick, controllable, and reversible way for both in vitro and in vivo studies.

## Materials and methods

Experimental details for synthesizing the intermediates of RC32 are described in Appendix SI (Supplementary Fig. S1). Original immunoblot analysis, a part of echocardiography result, cell lines (Supplementary Table S1), and antibodies (Supplementary Table S2) used in the study are listed in Appendix SI.

### Chemistry

To a solution of 4-((2-(2-(2-azidoethoxy)ethoxy)ethyl)amino)-2-(2,6-dioxopiperidin-3-yl)isoindoline-1,3-dione (0.43 g, 1 mmol), 40-*O*-(2-(prop-2-yn-1-yloxy)ethyl)-rapamycin (1.0 g, 1 mmol) in MeOH (100 mL) and tetrahydrofuran (20 mL), 1 M aqueous CuSO_4_ (3.0 mL, 3 mmol) and 1 M aqueous sodium ascorbate (1.5 mL, 1.5 mmol) were added. The mixture was stirred at room temperature overnight, then concentrated in vacuo. After extraction with dichloromethane (DCM) and brine, the crude material was purified by column chromatography on silica gel (elution with DCM-MeOH, 33:1) to make RC32 (0.71 g, 50%) a bright yellow solid.

^1^H NMR (400 MHz, CDCl_3_, ppm) δ 8.70 (s, 1H), 7.75 (s, 1H), 7.48 (t, *J* = 8.0 Hz, 1H), 7.09 (d, *J* = 6.8 Hz, 1H), 6.89 (d, *J* = 8.4 Hz, 1H), 6.50 (t, *J* = 4.9 Hz, 1H), 6.34 (dd, *J* = 16.6, 10.2 Hz, 1H), 6.13 (dd, *J* = 15.2, 10.2 Hz, 1H), 5.95 (d, *J* = 10.2 Hz, 1H), 5.55 (dd, *J* = 14.8, 9.2 Hz, 1H), 5.40 (d, *J* = 10.0 Hz, 1H), 5.26 (d, *J* = 6.0 Hz, 1H), 5.14 (q, *J* = 5.7 Hz, 1H), 4.89 (d, *J* = 5.7 Hz, 1H), 4.65 (s, 2H), 4.53 (s, 2H), 3.88 (t, *J* = 5.1 Hz, 2H), 3.75–3.62 (m, 12H), 3.46–3.36 (m, 12H), 3.12 (s, 3H), 2.86–2.66 (m, 4H), 2.33–2.30 (m, 2H), 2.10–2.08 (m, 2H), 2.10–2.08 (m, 4H), 1.74–1.72 (m, 5H), 1.64–1.45 (m, 10H), 1.48–1.42 (m, 4H), 1.30–1.19 (m, 9H), 1.07 (d, *J* = 6.8 Hz, 4H), 1.03 (d, *J* = 6.5 Hz, 4H), 0.97 (d, *J* = 6.5 Hz, 2H), 0.93 (d, *J* = 6.5 Hz, 2H), 0.89–0.82 (m, 8H). ^13^C NMR (100 MHz, CDCl_3_, ppm) δ 208.3, 192.9, 171.3, 169.5, 169.4, 168.7, 167.7, 166.8, 146.9, 140.1, 136.2, 136.1, 135.8, 133.7, 132.7, 130.3, 129.5, 126.7, 126.6, 116.8, 111.8, 110.5, 98.6, 85.0, 84.4, 83.2, 70.7, 70.6, 70.2, 69.7, 69.4, 69.3, 68.1, 67.3, 65.9, 64.7, 59.4, 58.0, 56.0, 51.4, 50.4, 49.0, 46.7, 44.3, 42.4, 41.6, 40.8, 40.3, 39.1, 38.4, 36.5, 35.2, 33.9, 33.3, 33.1, 31.9, 31.6, 31.3, 30.2, 27.3, 27.2, 25.7, 25.4, 22.9, 21.6, 20.8, 16.3, 16.1, 16.0, 15.4, 13.7, 13.4, 10.3. LRMS (ESI) calcd for C_75_H_107_N_7_O_20_ [M + H]^+^: 1427.72, found 1427.79.

The synthesis of P13IS was followed by our reported methods^31^.

^1^H NMR (400 MHz, CDCl_3_, ppm) δ 8.38 (s, 1H), 7.62–7.37 (m, 6H), 7.13–7.07 (m, 6H), 6.89 (d, *J* = 8.0 Hz, 1H), 6.53–6.50 (m, 1H), 4.91–4.79 (m, 2H). 4.51–4.42 (m, 2H), 3.69 (s, 2H), 3.62–3.46 (m, 10H), 2.74 (m, 4H), 2.42–2.00 (m, 10H), 1.66 (m, 2H). ^13^C NMR (100 MHz, CDCl_3_, ppm) δ 169.5, 167.8, 158.6, 156.4, 146.8, 136.2, 132.7, 130.1, 127.7, 124.1, 119.6, 119.3, 116.9, 111.8, 110.4, 70.9, 70.6, 70.5, 70.0, 69.8, 69.4, 69.3, 66.0, 53.6, 50.3, 49.0, 42.3, 41.9, 29.8, 25.2, 25.0, 22.9. LRMS (ESI) calcd for C_47_H_5_0N_12_O_8_ [M + H]^+^: 912.00, found 911.91.

### Cell cultures and FKBP12 protein degradation assay

All the complete growth media were supplemented with 10% fetal bovine serum and 1% penicillin–streptomycin (100×). Human Jurkat, Ramos, and C4–2 cells were cultured in RPMI 1640; chicken DT40 cells were cultured in RPMI 1640 with 5% chicken serum; human MDA-MB-453, MCF7, and HeLa cells, mouse MIN6 cells, and rat H9C2 cells were cultured in Dulbecco’s modified Eagle’s medium; and human PA-1 cells were cultured in McCoy's 5A (modified) medium. All the cells were grown at 37 °C under 5% CO_2_. To investigate the cellular degradation of FKBP12, adherent cells were seeded in a 6-well plate at a density of 2 × 10^5^, allowed to attach overnight, and incubated with the indicated compounds for 12 h. Cells in suspension were seeded in a 6-well plate at a density of 5 × 10^5^, left overnight, and incubated with the indicated compounds for 12 h. Compounds were dissolved in dimethylsulfoxide (DMSO) for storage (10 mM). When indicated, cells were pretreated with 0.4 μM bortezomib, 0.4 μM carfilzomib, 10 μM pomalidomide, or 10 μM rapamycin for 3 h and then treated with RC32 (10 nM) for 2 h. Regarding washout studies, after RC32 (1 µM) treatment for 12 h, cells were washed three times with phosphate-buffered saline and incubated with complete medium for the indicated time before harvesting. Cells were also routinely tested to ensure that they were negative for mycoplasma. All the cells were lysed on ice for 20 min with RIPA lysis buffer containing 1% protease inhibitor (100×) and 1% phenylmethylsulfonyl fluoride (PMSF, 100×), followed by addition of SDS loading buffer (4×) and heating for 10 min at 100 °C.

### Antibodies

Anti-FKBP12 antibodies were from Santa Cruz (sc-133067, 1:750 dilution). Anti-BTK antibodies were from Absin (abs135992, 1:1000 dilution). Anti-actin antibodies were from ABclonal (AC004, 1:10,000); anti-GAPDH antibodies were from Jiangsu Kangwei Biotechnology (CW0100M, 1:2000); anti-tubulin antibodies were from Beyotime (AT819, 1:1000); and anti-mouse IgG-horseradish peroxidase (HRP) (7076S) and anti-rabbit IgG-HRP (7074S) were from Cell Signaling Technology and used at 1:5000.

### Immunoblotting

Total protein concentrations in cell lysates were determined using a Solarbio BCA kit. Cell lysates were separated on 5–15% SDS-PAGE gels and transferred onto PVDF membranes (Merck Millipore) that were then blocked with 5% non-fat milk in TBST (0.05% Tween-20 in Tris-buffered Saline) before overnight incubation with the indicated antibodies at 4 °C. After incubation with the appropriate HRP-conjugated secondary antibodies, the bands were visualized by enhanced chemiluminescence (BeyoECL Plus). The band intensity was quantified with ImageJ software. Statistical analyses of tissue densitometry values were performed with GraphPad Prism software using the indicated statistical tests.

### Animals

The compound RC32 and P13IS were dissolved in 0.9% NaCl (aq.) containing 5% DMSO and 10% castor oil. The vehicle group was given 0.9% NaCl (aq.) containing 5% DMSO and 10% castor oil. Different tissues were harvested from animals and flash frozen in liquid nitrogen for further analysis. Small pieces (10–20 mg) of the collected tissues were lysed with RIPA lysis buffer containing 1% PMSF and 1% protease inhibitor on ice for 30 min. Then the lysate buffer was centrifuged for 10 min at 4 °C and the supernatant was collected to centrifuge tubes with the addition of SDS loading buffer.

All mouse and rat procedures were performed in adherence with the Guide for the Care and Use of Laboratory Animals published by the U.S. National Institutes of Health (NIH Publication No. 85-23, revised 1996), and with the approval of the Institute of Biophysics Committee for Animal Care. Mice and rats were anesthetized with chloral hydrate before sacrifice. To measure the FKBP12 degradation and recovery in ICR mice after i.p. injection, males and females were randomized into two groups. One group served as a control for the vehicle, while the other group was given two doses of RC32 in 1 day (200 µL, 30 mg/kg, i.p., every 12 h). Mice were sacrificed 12 h after the final dose. Male mice were used for FKBP12 protein recovery studies. After all the mice were given two doses of vehicle or RC32 in 1 day (30 mg/kg, i.p., every 12 h), they were fed until the indicated day of sacrifice. To assess FKBP12 degradation in ICR mice after i.c.v. administration, each male ICR mouse was given 0.15 mg RC32 in 3 µL DMSO or vehicle (3 µL DMSO) by a single left ventricular injection. Mice were sacrificed 12 h later, and the brain and heart were harvested for further analysis. To determine FKBP12 degradation through oral administration in FVB mice, males were given RC32 with food (60 mg/kg, twice per day for 2 days) and sacrificed 12 h after the final dose. For Ca^2+^ spark determination and echocardiography in ICR mice, all the mice used were injected i.p. with RC32 for 30 days (30 mg/kg, twice a day for the first 2 days and once a day for the remaining 28 days). Single ventricular myocytes from adult mice (6–10 months old) were prepared as previously described^1^ (Supplementary references) and incubated with 10 mM Fluo-4 AM (Molecular Probes) for 15 min at room temperature, placed in a recording chamber mounted on an inverted microscope (Zeiss 880), and perfused for 40 min at room temperature with extracellular fluid (in mM: 140 NaCl, 5.4 KCl, 1.8 CaCl_2_, 1.2 MgCl_2_, 10 HEPES pH 7.4, and 10 glucose). Fluo-4 fluorescence was recorded using a laser scanning confocal head (Bio-Rad Laboratories) attached to an inverted microscope (Zeiss 880) with a ×64 oil-immersion objective. Cells were excited with 488-nm light from a krypton–argon laser. Line-scan images were recorded at an interval of 921.6 μs per line. The images were analyzed using SparkMaster in ImageJ. Fluorescence profiles were constructed by averaging two pixels bisecting a Ca^2+^ spark at each time point in the scan from the line-scan images using ImageJ.

Echocardiographs were recorded using a 30-MHz probe and a Vevo 770 Ultrasound machine (VisualSonics, Toronto, ON, Canada). The Vevo 770 was equipped with ECG-gated kilohertz visualization software. Mice were anesthetized with isoflurane and placed on a temperature-controlled plate (37 ± 1 °C) and the ECG was recorded. Measurements and calculations were performed blinded. To investigate FKBP12 degradation in Sprague–Dawley rats, male and female rats were randomized into two groups. One group served as a control for the vehicle, while the other group was given two doses of RC32 in 1 day (200 µL, 20 mg/kg, i.p., every 12 h). Rats were sacrificed 12 h after the final dose.

The Bama pigs used in this study had ad libitum access to a commercial pig diet (nutrient levels according to the United States National Research Council) and water throughout the experimental period. Protocols for all experiments were approved by the Animal Care and Use Committee of the Institute of Zoology, Chinese Academy of Sciences, China. Pigs were anesthetized (5 kg/mL, intravenous injection) with xylazine before sacrifice. Male and female pigs were randomized into two groups. One group served as a control for dosing vehicle, while the other group was given RC32 twice a day for 2 days (20 mL, 8 mg/kg, i.p., every 12 h). Pigs were sacrificed 12 h after the final dose.

The experimental procedures for rhesus monkeys were approved by the Animal Care and Use Committee of Peking University and were carried out in accord with the principles of laboratory animal care of the National Academy of Sciences/National Research Council. The monkeys were housed individually in cages in the Laboratory Animal Center of Peking University, with a 12-h light/dark cycle, a temperature range of 18–24 °C, and humidity 40–70%. The monkeys had free access to water and were fed ad libitum with monkey chow (Beijing HFK Bio-Technology Co. Ltd., China), which contained 7–10% crude fat, 16–20% crude protein, and 55–65% crude carbohydrate. Blood samples were taken and blood pressure was recorded after a 14–16 h fast, then the monkeys were anesthetized with ketamine (ketamine hydrochloride injection, 2 mL, 0.1 g, Fujian Gutian, China; ~10 mg/kg body weight). The body weight was measured using an electronic scale. Each blood sample was taken from a hind limb vein through an intravenous cannula, and the plasma was used for biochemical tests. Blood pressure was recorded at 5-min intervals for 40 min with a monitor (iPM-10, Mindray Medical International Ltd., Shenzhen, China) on one forearm while the monkey was supine. To assess FKBP12 degradation and confirm the efficacy of FKBP12 knockdown, 2 monkeys were euthanized after 3 days of administration of RC32 (8 mg/kg, i.p., twice a day). The monkeys were anesthetized with ketamine (~10 mg/kg body weight) and isoflurane (3–5%), and then exsanguinated through the aorta. Samples of different organs were quick-frozen in liquid nitrogen and stored at −80 °C until further measurement. For echocardiography, 6 monkeys were randomized into two groups and given RC32 for 15 days (8 mg/kg, i.p., twice a day for 3 days, and 8 mg/kg, i.p., once a day for 12 days). To obtain biopsies, monkeys were anesthetized with ketamine (~10 mg/kg body weight) and maintained with isoflurane (1–3%) during the biopsy. Buprenorphine (0.01 mg/kg body weight) was used before and after biopsy for analgesia. Skin and muscle were taken from the lateral femoral region. Fat tissue was taken from the lower abdominal region.

The experimental procedures for detection of BTK and FKBP12 degradation in mice were similar.

### Statistics and reproducibility

All experiments were repeated at least three times and all data are presented as the mean ± SEM. Statistical calculations were analyzed using GraphPad Prism Version 6. A two-tailed unpaired Student’s *t*-test was used to analyze Western blots, fluorescence quantitative PCR, and Ca^2+^ sparks. For echocardiography of the mice and monkeys, two-way ANOVA was performed with appropriate multiple comparisons. All statistics are representative of biological replicates (*p *<* 0.05, *p *<* 0.01, n.s. not significant).

## Supplementary information

Supplementary Information

## Supplementary information

**Supplementary Information** accompanies the paper at (10.1038/s41421-018-0079-1).
