# Supplementary material for: A chemical approach for global protein knockdown from mice to non-human primates
Source: Cell Discov. 2019 Feb 5;5:10. doi: 10.1038/s41421-018-0079-1 (PMC6361926; doi:10.1038/s41421-018-0079-1)
Supplement: Supplementary file 1 — Supplementary Information [file 41421_2018_79_MOESM1_ESM.pdf]

Supplementary Information for

A Chemical Approach for Global Protein Knockdown from Mice to Non-human Primates

Xiuyun Sun[1,2,9], Jun Wang[3,4,9], Xia Yao[1,9], Wen Zheng[5,6], Yang Mao[3,4], Tianlong Lan[1], Liguo Wang[1], Yonghui Sun[1], Xinyi Zhang[3,4], Qiuye Zhao[1], Jianguo Zhao[7], Rui-Ping Xiao[8,9], Xiuqin Zhang[5,6*], Guangju Ji[3*], Yu Rao[1*]
Corresponding author: Xiuqin Zhang, Guangju Ji, Yu Rao
Email: zhangxq@pku.edu.cn; gj28@ibp.ac.cn; yrao@tsinghua.edu.cn

This file includes:

Supplementary text
Figs. S1 to S14
Tables S1 to S2
References for SI reference citations

Other supplementary materials for this manuscript include the following:

^{1}H NMR and ^{13}C NMR spectrum for **RC32**
^{1}H NMR and ^{13}C NMR spectrum for **P13IS**

Supplementary Information Text

Materials and Methods. The synthesis of intermediates for RC32 and detailed immunoblot results were descried in this part. In addition, the cell lines and antibodies used in the study were listed in *SI Appendix*. The synthesis of P13IS was followed by our reported methods.

The synthesis and characterization of rapamycin-based PROTACs targeting FKBP12 (RC32)

To a solution of 2-(prop-2-yn-1-yloxy)ethanol (19.5 mL, 0.20 mol) in CH_2Cl_2 (150 mL) was added 2,6-lutidine (32.6 mL, 0.28 mol) followed by trifluoromethanesulfonic anhydride (43.2 mL, 0.26 mol) at -50 ℃ under argon atmosphere. The mixture was stirred at -10 ℃ for 2 h. Then it was extracted by DCM and brine. The organic layer was collected and dried over anhydrous Na_2SO_4. The insoluble was filtered and the filtrate was evaporated in vacuo. The crude material was purified by column chromatography on silica gel (eluting with petroleum ether-ethyl acetate, 33:1). The desired fractions were combined and evaporated under vacuum to give the titled compound **S2** (20 g, 43%) as a colorless oil.

To a solution of rapamycin (0.91 g, 1 mmol) in toluene (1 mL) were added a solution of 2-(prop-2-yn-1-yloxy)ethyl trifluoromethanesulfonate (**S2**, 1.74 g, 7.5 mmol) in toluene (1 mL) and N,N-diisopropyl-N-ethylamine (8.2 mL, 50 mmol) at 0 ℃. The mixture was warmed to 60 ℃ and stirred for 30 min. An additional amount of 2-(prop-2-yn-1-yloxy)ethyl trifluoromethanesulfonate (1.74 g, 7.5 mmol) in toluene (1 mL) was added and the mixture was stirred at 60 ℃. After 30 min, the final portion of 2-(prop-2-yn-1-yloxy)ethyl trifluoromethanesulfonate (1.74 g, 7.5 mmol) in toluene (1 mL) was added. The mixture was stirred at 60 ℃ for additional 1 h. It was then cooled and partitioned between DCM and brine. The organic layer was washed with brine and dried over anhydrous Na_2SO_4. The insoluble was filtered off and the filtrate was evaporated in vacuo. The crude material was purified by column chromatography on silica gel (eluting with petroleum ether-ethyl acetate, 1:1). Desired fractions were combined and evaporated under vacuum to give compound **S3** (0.5 g, 50%) as a white solid.

To a solution of 4-((2-(2-(2-azidoethoxy)ethoxy)ethyl)amino)-2-(2,6-dioxopiperidin-3-yl)isoindoline-1,3-dione (**S3**, 0.43 g, 1 mmol) and 40-O-(2-(prop-2-yn-1-yloxy)ethyl)-rapamycin (1.0 g, 1 mmol) in MeOH (100 mL) and THF (20 mL) were added 1 M aqueous $CuSO_4$ solution (3.0 mL, 3 mmol) and 1 M aqueous sodium ascorbate solution (1.5 mL, 1.5 mmol). The mixture was stirred at room temperature overnight. It was then concentrated in vacuo. After extraction with DCM and brine, the crude material was purified by column chromatography on silica gel (eluting with DCM-MeOH, 33:1) to give **RC32** (0.71 g, 50%) as a bright yellow solid.

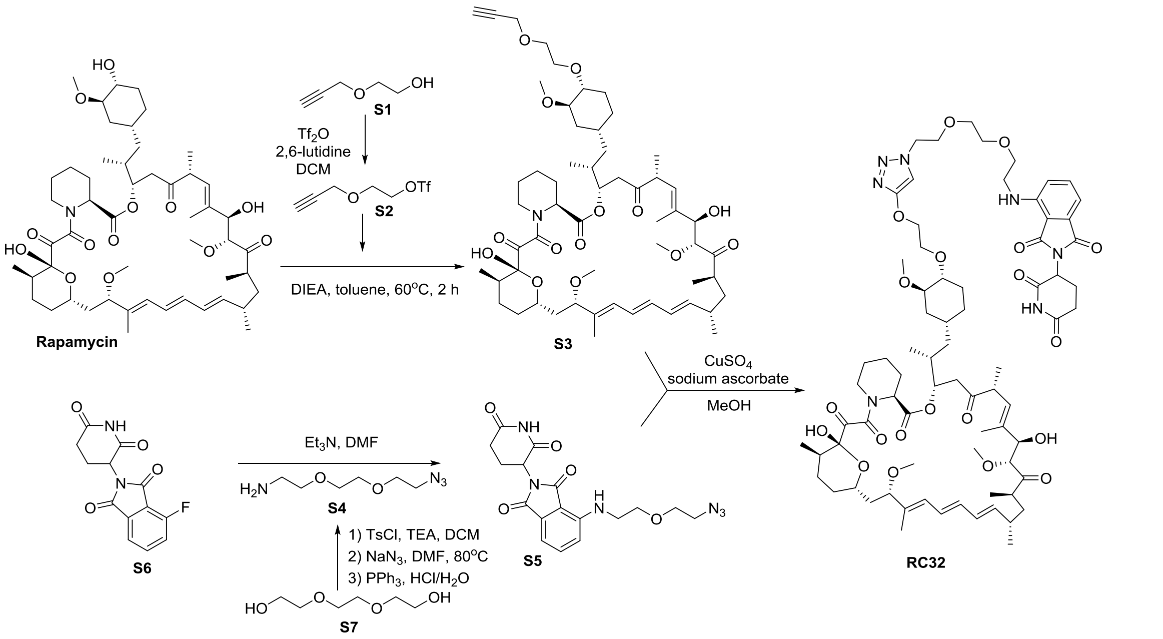

Fig. S1. The synthetic procedure of **RC32**.

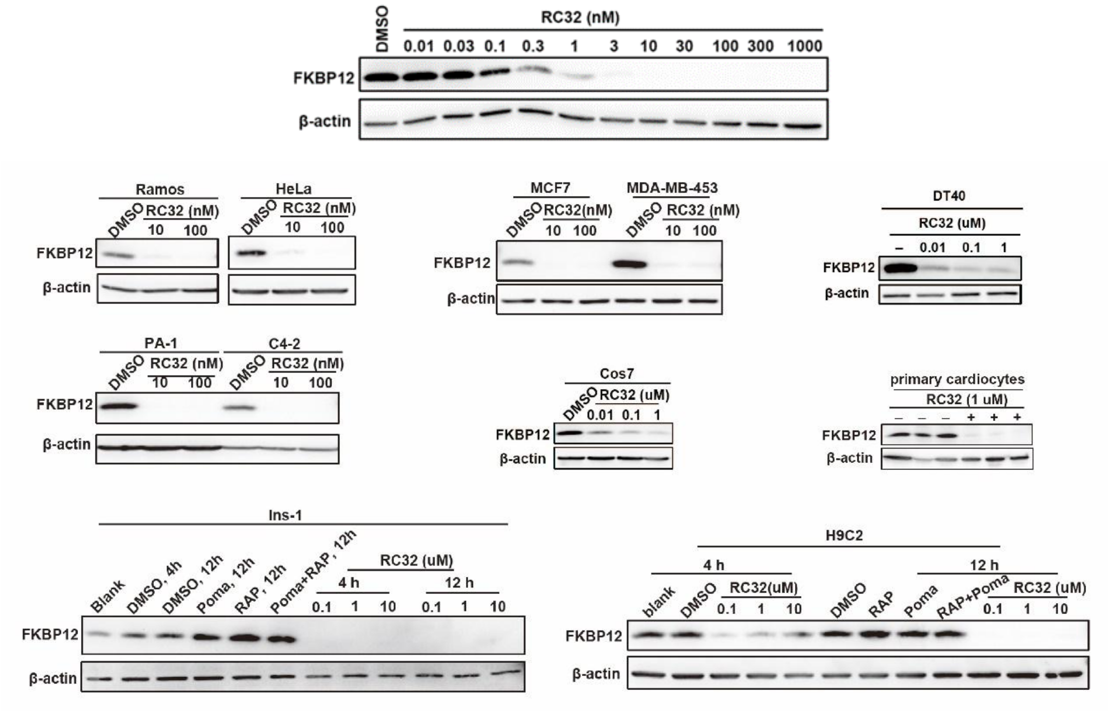

Fig. S2. DC$_{50}$ of **RC32** in jurkat cells after 12 h treatment and its degrading efficiency in different cell lines and primary cardiocytes after 12 h treatment.

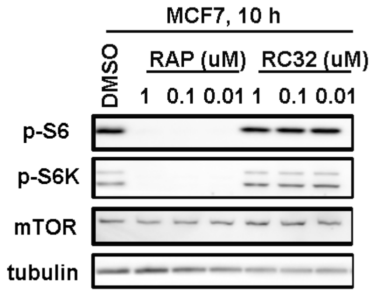

Fig. S3. The effect on mTOR pathway of **RC32** in MCF7 after treating for10 h.

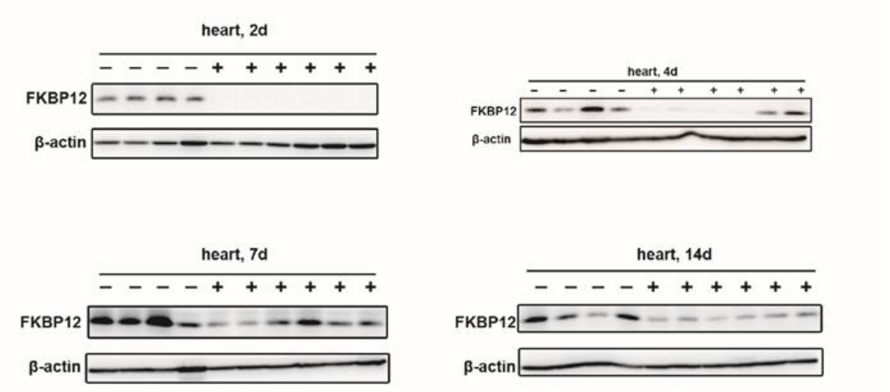

Fig. S4. The FKBP12 protein recovery in the heart of RC32-treated ICR mice (30 mg/kg, i.p., twice a day for 1 day) after withdrawal of RC32.

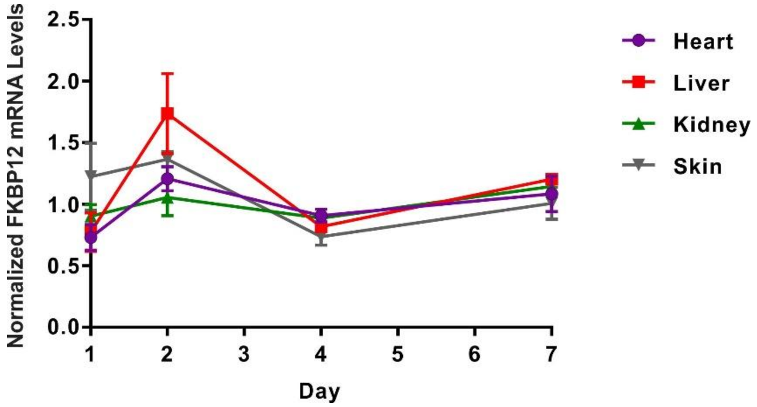

Fig. S5. The mRNA level at different days of RC32-treated ICR mice (30 mg/kg, i.p., twice a day for 1 day) in the recovery experiment. The RC32 was administrated in day 1 and withdrawn since day 2.

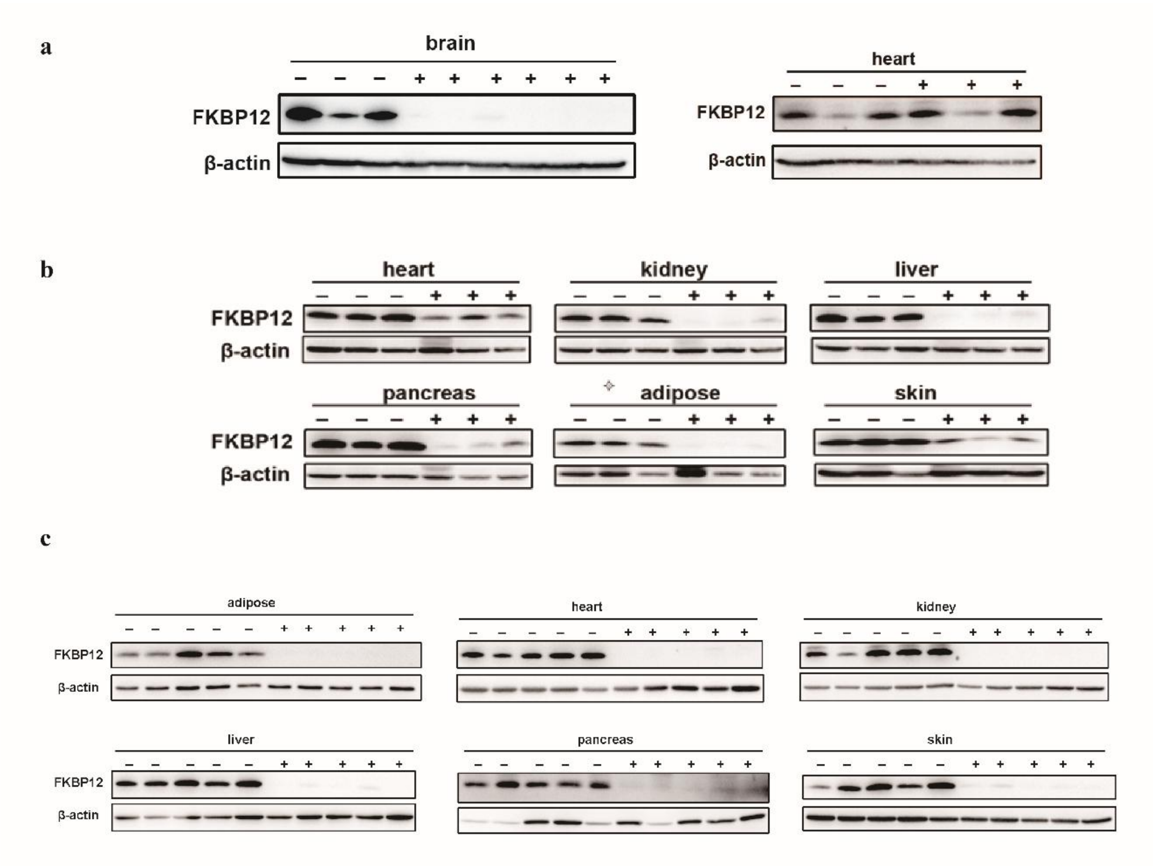

Fig. S6. The FKBP12 protein level of mice and rats. (**a**) FKBP12 level of brain and heart in **RC32-**treated mice through i.c.v. (a single injection, 0.2 mg in 2 μL). (**b**) FKBP12 level in **RC32-**treated mice through oral administration (60 mg/kg, twice a day for 1 day). (**c**) FKBP12 level in **RC32-**treated SD rats through i.p. (20 mg/kg, i.p., twice per day for 1 day).

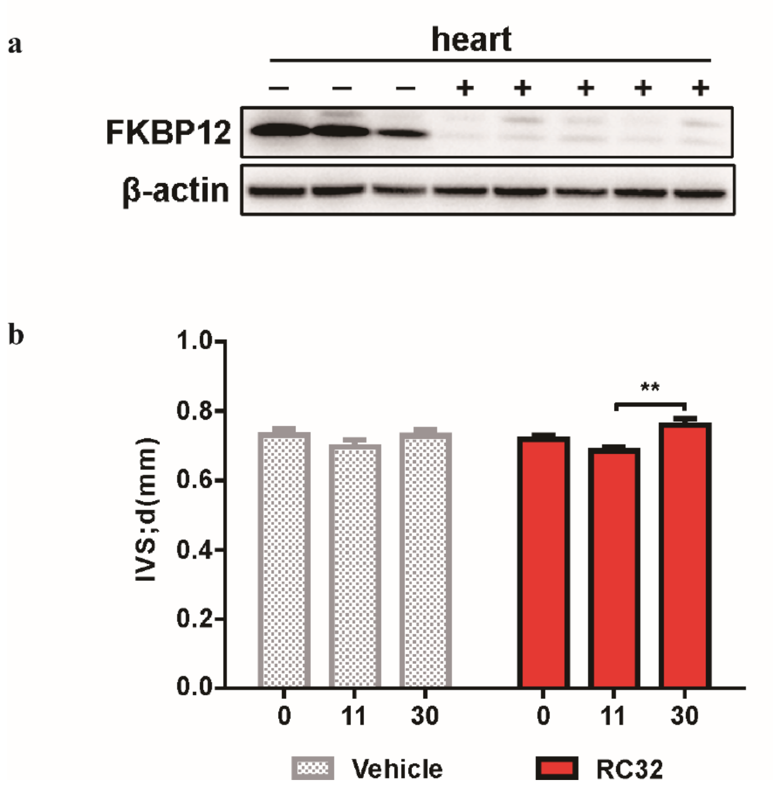

Fig. S7. The FKBP12 degradation in the heart of ICR mice (**a**) after 30 days administrations (30 mg/kg, i.p., twice a day for 2 days and once a day for 28 days) and their IVS;d results (**b**). When compared to the vehicle group, IVS in the RC32-treated group showed decrease at day 11. However, there was no statistical significance when the IVS results between two groups were analyzed statistically.

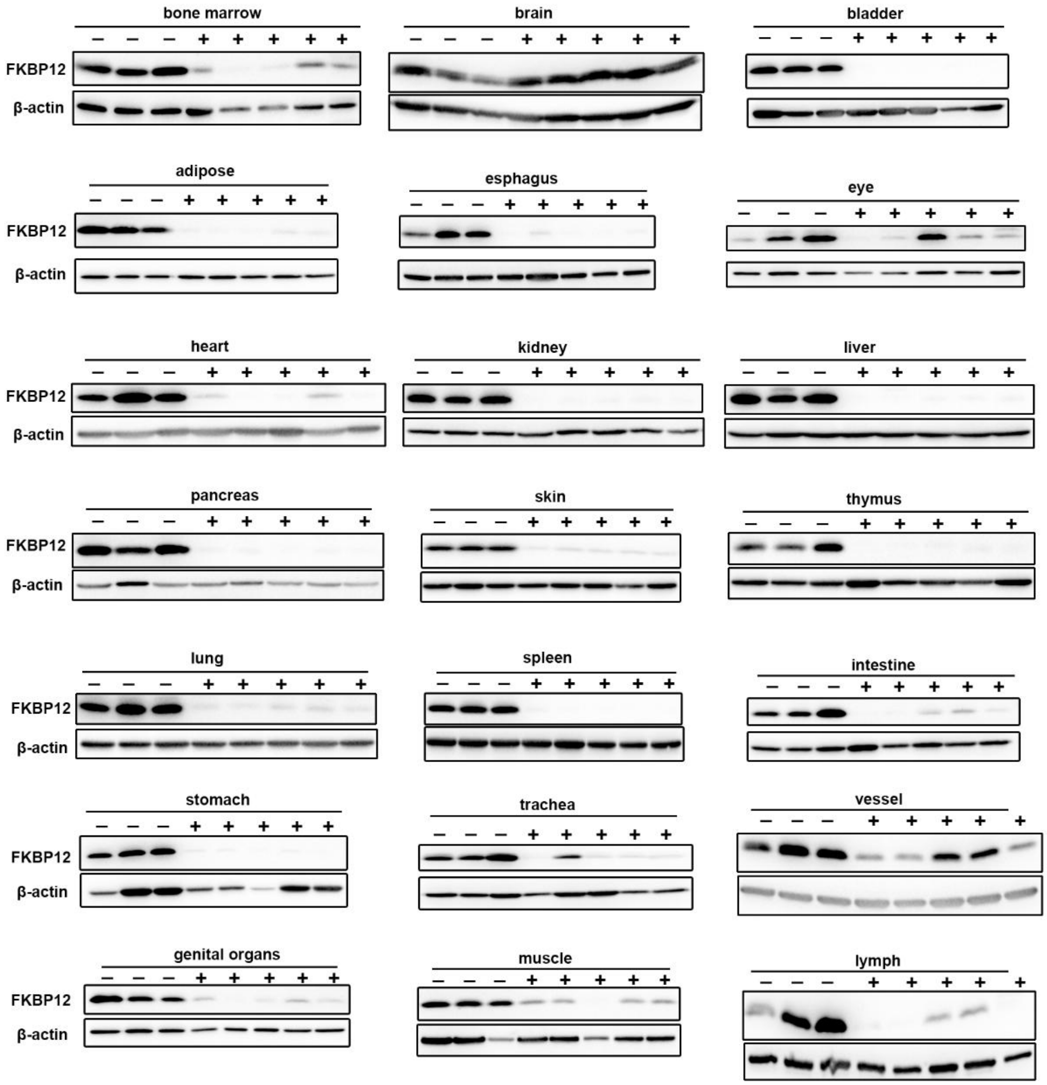

Fig. S8. The FKBP12 protein level in **RC32** treated-bama pigs through i.p. (8 mg/kg, i.p., and twice a day for 2 days).

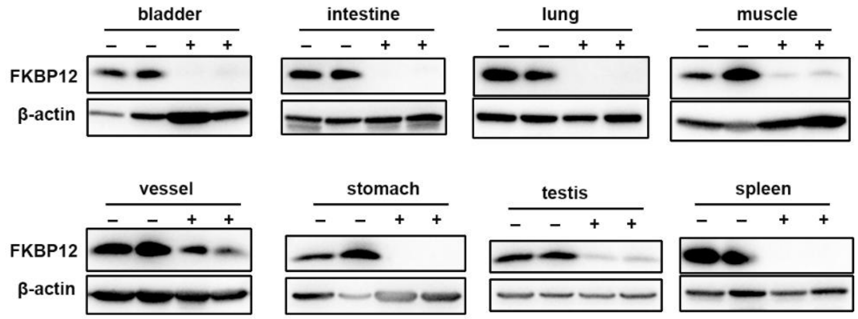

Fig. S9. The FKBP12 protein level in **RC32**-treated rhesus monkeys through i.p. (8 mg/kg, i.p., and twice a day for 3 days).

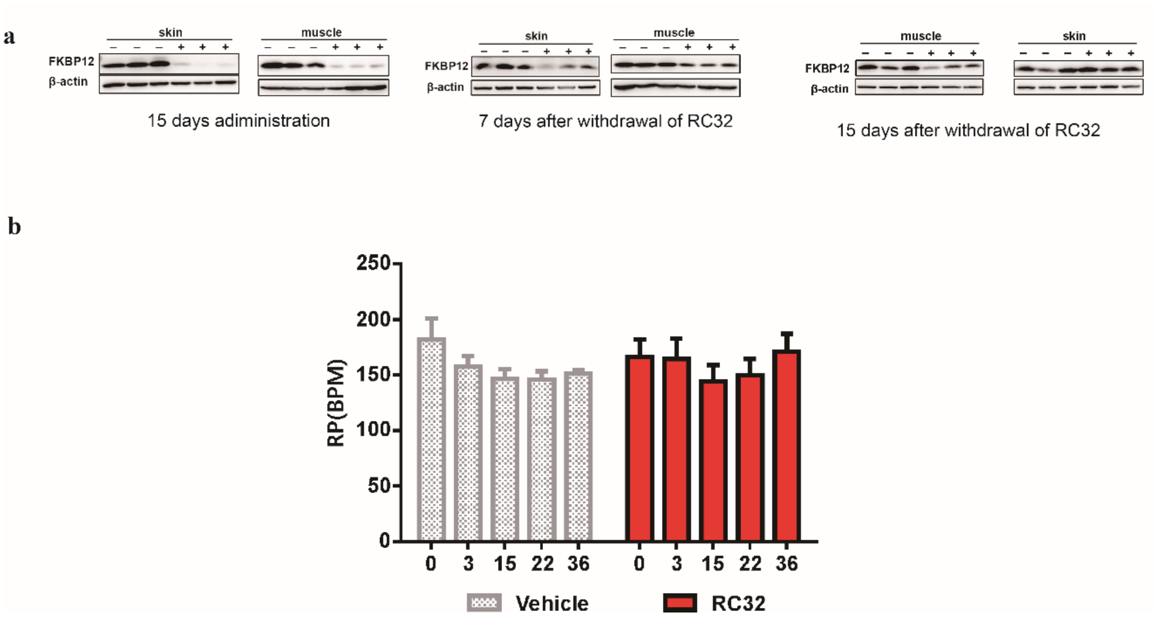

Fig. S10. The FKBP12 protein level (**a**) and Pulse rate (PR, **b**) in the **RC32**-treated group (8 mg/kg, i.p., twice a day for 3 days, and 8 mg/kg, i.p., once a day for 12 days) compared with the vehicle group and recovery after the withdrawal of the PROTAC.

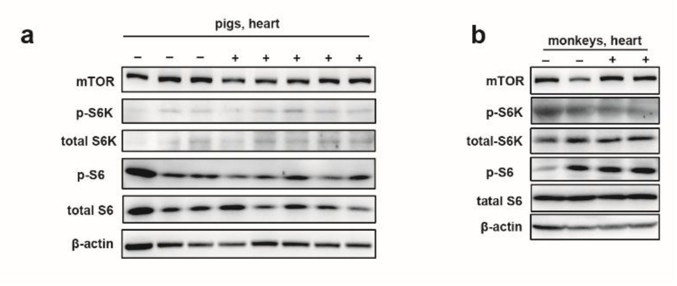

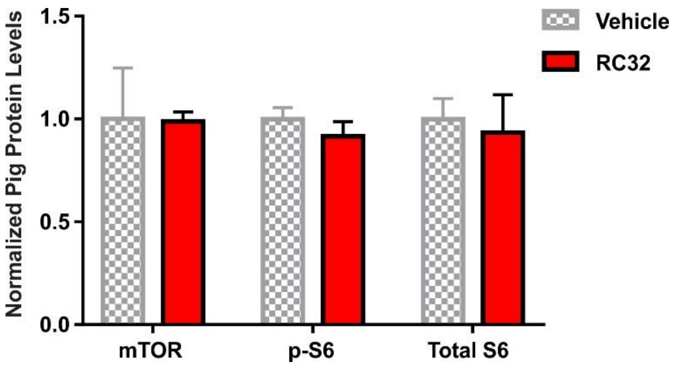

Fig. S11. The mTOR protein levels in the RC32-treated pigs (8 mg/kg, i.p., and twice a day for 2 days) (**a**) and rhesus monkeys (8 mg/kg, i.p., and twice a day for 3 days) (**b**) compared with the vehicle group. Due to the poor imaging of p-S6K and S6K in pigs, we did not analyze these two proteins statistically. Meanwhile, it was inaccurate to analyze the samples of rhesus monkeys because only two samples were performed.

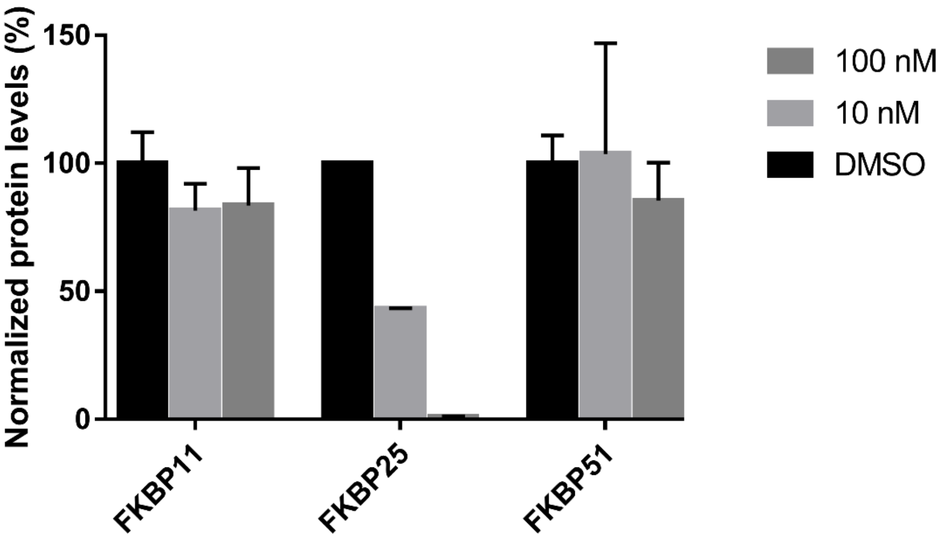

Fig. S12. The degrading selectivity of RC32 in Jurkat cells after 12 h treatment. The data are demonstrated as the mean ratio ± SEM and represent three independent experiments.

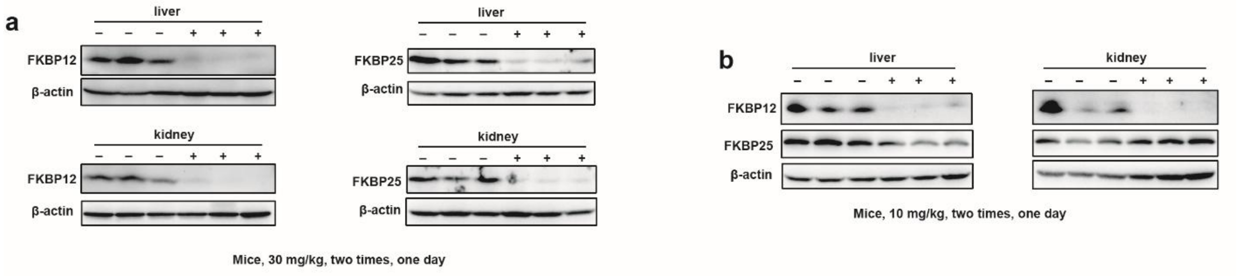

Fig. S13. The FKBP12 and FKBP25 levels in liver and kidney of RC32-treated mice with different dosage. (**a**) The two protein levels in RC32-treated mice with 30 mg/kg (twice a day for one day). (**b**) The two protein levels in RC32-treated mice with 10 mg/kg (twice a day for one day).

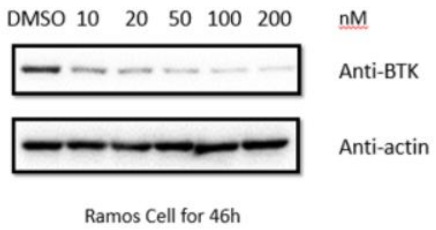

Fig. S14. The degrading efficiency of P13IS in ramos cells after treating for 46 h.

Table S1. Cell lines and authentication.

Cell line	Source	STR score
Ramos	ATCC	100
MCF7	ATCC	100
HeLa	ATCC	100
C4-2	ATCC	100
MDA-MB-453	ATCC	100
PA-1	ATCC	100
MIN6	ATCC	100
H9C2	ATCC	100
Cos7	ATCC	100
DT40	ATCC	100

Table S2. Antibodies used in this study.

Antibody	Species	Source	Catalog Number
FKBP12	Mouse	Santa Cruz	sc-133067
Actin	Mouse	ABclonal	AC004
GAPDH	Mouse	Jiangsu Kangwei Biotechnology	CW0100M
Tubulin	Mouse	Beyotime	AT819
IgG-HRP	Mouse	Cell Signaling	7076S
IgG-HRP	Rabbit	Cell Signaling	7074S
FKBP12.6	Mouse	Santa Cruz	sc-376135
FKBP11	Mouse	Santa Cruz	sc-398700
FKBP25	Mouse	Santa Cruz	sc-374357
FKBP51	Mouse	Santa Cruz	sc-271547
FKBP51	Rabbit	Abcam	ab126715

The amino acid sequence of FKBP12 in different species.
Human
MGVQVETISPGDGRTFPKRGQTCVVHYTGMLEDGKKFDSSRDRNKPFKFMLGKQEVIRGME
EGVAQMSVGQRAKLTISPDYAYGATGHPGIIPPHATLVFDVELLKLE

Mouse
MGVQVETISPGDGRTFPKRGQTCVVHYTGMLEDGKKFDSSRDRNKPFKFTLGKQEVIRGME
EGVAQMSVGQRAKLIISPDYAYGATGHPGIIPPHATLVFDVELLKLE

Rat
MGVQVETISPGDGRTFPKRGQTCVVHYTGMLEDGKKFDSSRDRNKPFKFTLGKQEVIRGME
EGVAQMSVGQRAKLIISPDYAYGATGHPGIIPPHATLVFDVELLKLE

Chicken
MGVHVETIAPGDGRTFPKRGQTCVVHYTGMLEDGKKFDSSRDRNKPFKFVMGKQEVIRGME
EGVAQMSVGQRAKMTISPDYAYGSTGHPGIIPPNATLIFDVELMKLE

Pig
MGVQVETISPGDGRTFPKRGQTCVVHYTGMLEDGKKFDSSRDRNKPFKFVLGKQEVIRGME
EGVAQMSVGQRAKLTISPDYAYGATGHPGIIPPNATLVFDVELLKLE

Monkey
MGVQVETISPGDGRTFPKRGQTCVVHYTGMLEDGKKFDSSRDRNKPFKFMLGKQEVIRGME
EGVAQMSVGQRAKLTISPDYAYGATGHPGIIPPHATLVFDVELLKLE

References

1. Lehnart SE, Wehrens XH, Marks AR (2005) Defective ryanodine receptor interdomain interactions may contribute to intracellular Ca^{2+} leak: a novel therapeutic target in heart failure. *Circulation* 111: 3342–3346.

NMR data

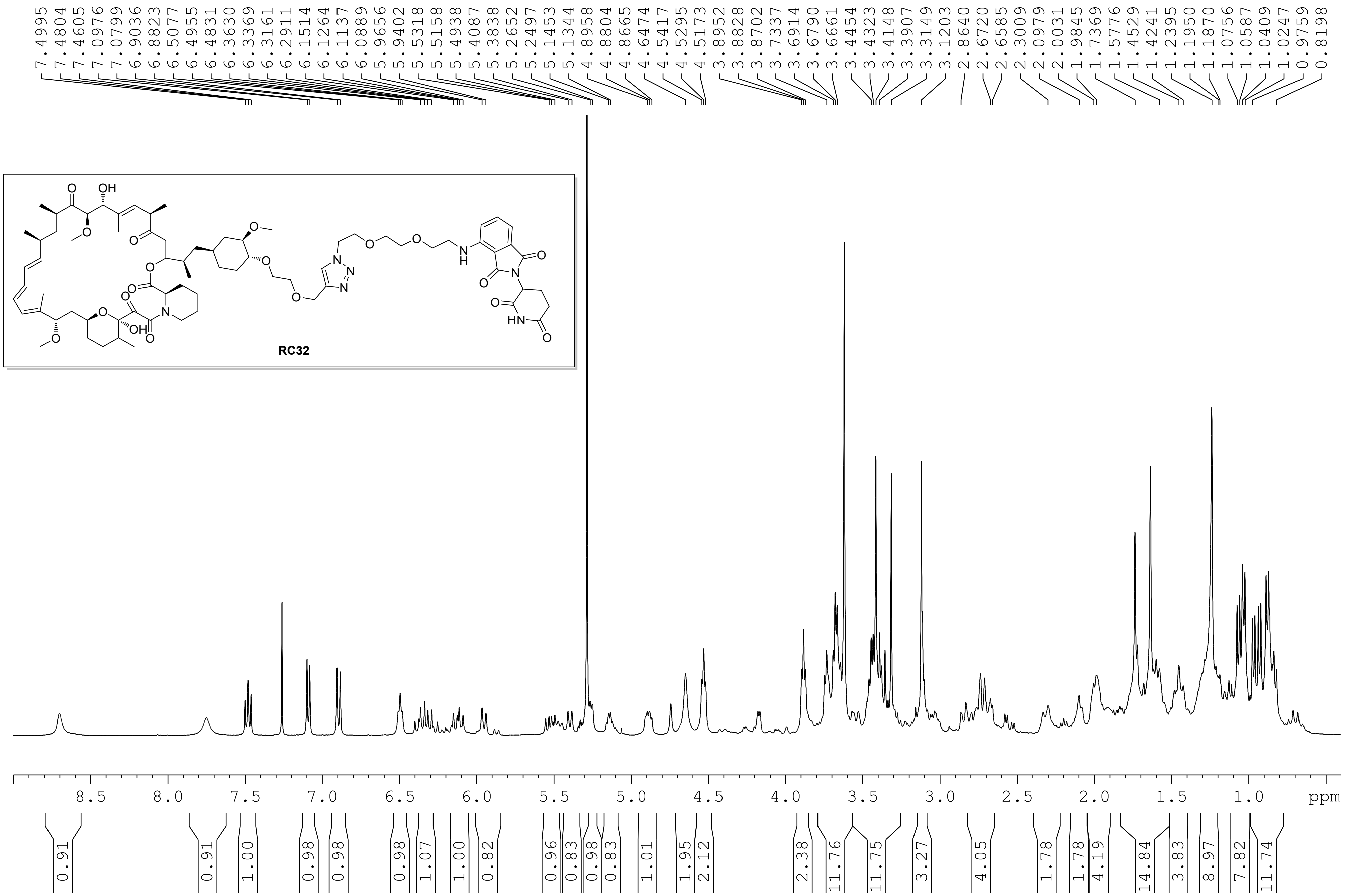

7.4995
7.4804
7.4605
7.0976
7.0799
6.9036
6.8823
6.5077
6.4955
6.4831
6.3630
6.3369
6.3161
6.2911
6.1514
6.1264
6.1137
6.0889
5.9656
5.9402
5.5318
5.5158
5.4938
5.4087
5.3838
5.2652
5.2497
5.1453
5.1344
4.8958
4.8804
4.8665
4.6474
4.5417
4.5295
4.5173
3.8952
3.8828
3.8702
3.7337
3.6914
3.6790
3.6661
3.4454
3.4323
3.4148
3.3907
3.3149
3.1203
2.8640
2.6720
2.6585
2.3009
2.0979
2.0031
1.9845
1.7369
1.5776
1.4529
1.4241
1.2395
1.1950
1.1870
1.0756
1.0587
1.0409
1.0247
0.9759
0.8198
RC32
8.5 8.0 7.5 7.0 6.5 6.0 5.5 5.0 4.5 4.0 3.5 3.0 2.5 2.0 1.5 1.0 ppm
0.91
0.91
1.00
0.98
0.98
0.98
1.07
1.00
0.82
0.96
0.83
0.98
0.83
1.01
1.95
2.12
2.38
11.76
11.75
3.27
4.05
1.78
1.78
4.19
14.84
3.83
8.97
7.82
11.74

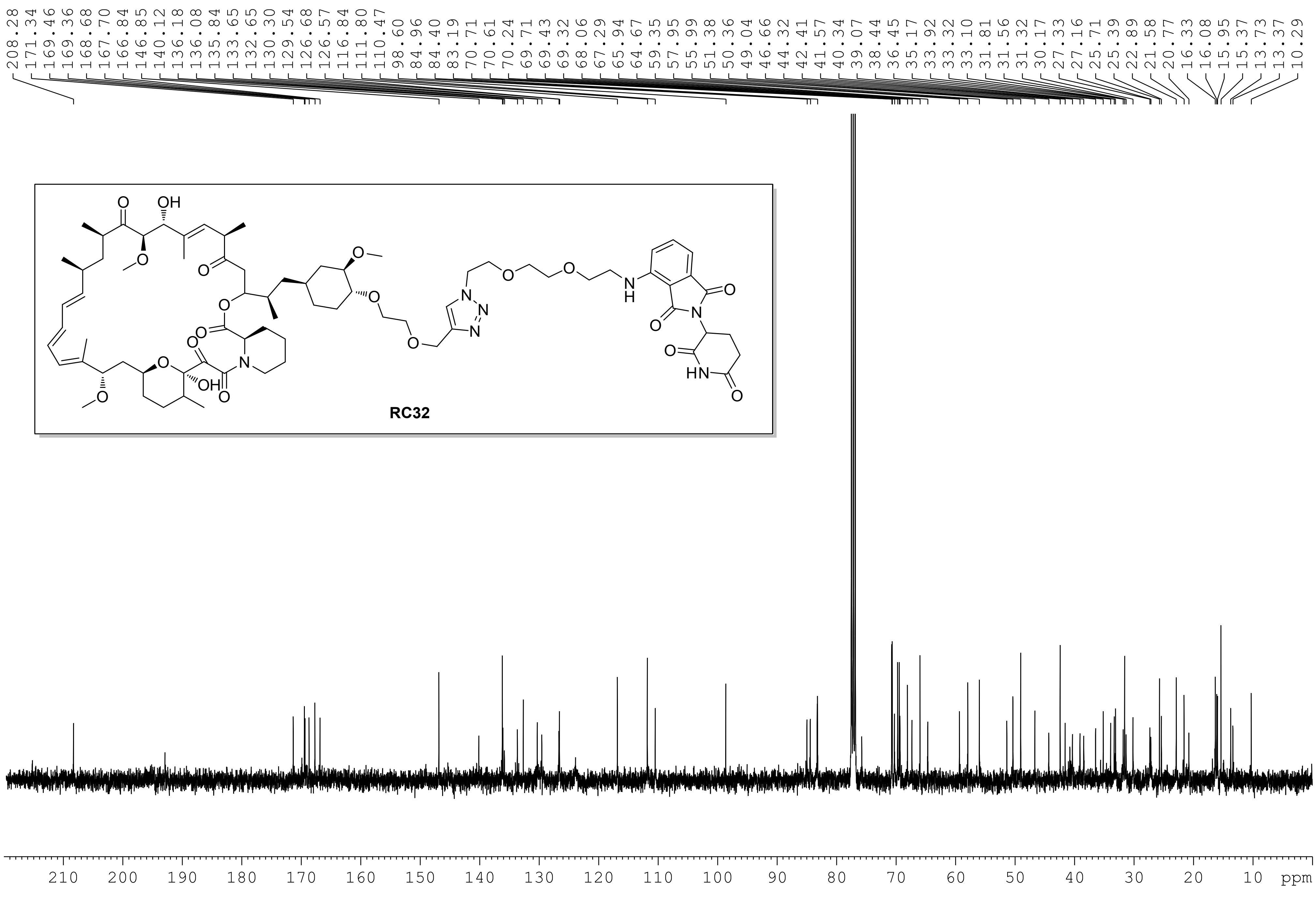
208.28
171.34
169.46
169.36
168.68
167.70
166.84
146.85
140.12
136.18
136.08
135.84
133.65
132.65
130.30
129.54
126.68
126.57
116.84
111.80
110.47
98.60
84.96
84.40
83.19
70.71
70.61
70.24
69.71
69.43
69.32
68.06
67.29
65.94
64.67
59.35
57.95
55.99
51.38
50.36
49.04
46.66
44.32
42.41
41.57
40.34
39.07
38.44
36.45
35.17
33.92
33.32
33.10
31.81
31.56
31.32
30.17
27.33
27.16
25.71
25.39
22.89
21.58
20.77
16.33
16.08
15.95
15.37
13.73
13.37
10.29
RC32
210 200 190 180 170 160 150 140 130 120 110 100 90 80 70 60 50 40 30 20 10 ppm

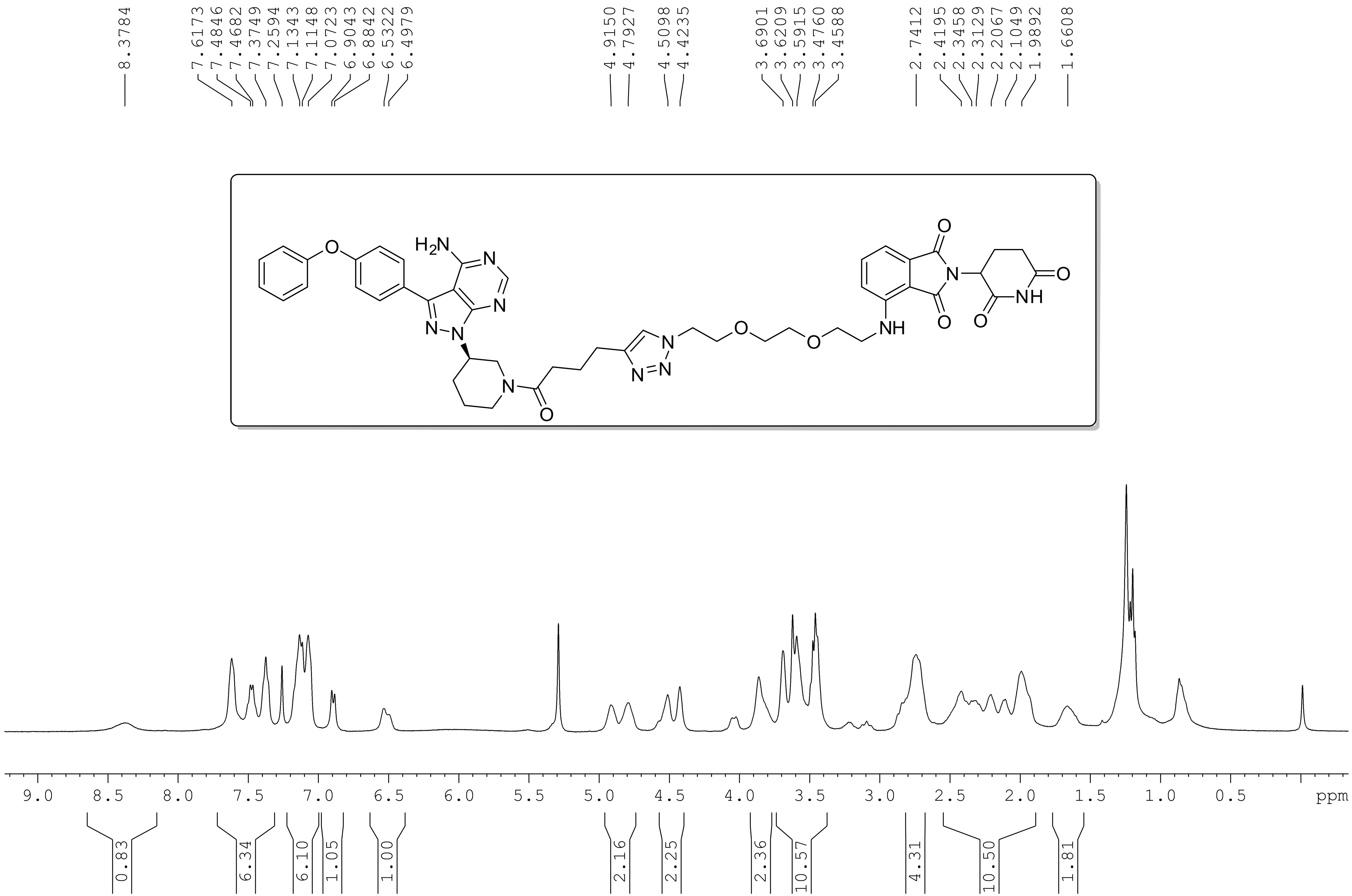
8.3784
7.6173
7.4846
7.4682
7.3749
7.2594
7.1343
7.1148
7.0723
6.9043
6.8842
6.5322
6.4979
4.9150
4.7927
4.5098
4.4235
3.6901
3.6209
3.5915
3.4760
3.4588
2.7412
2.4195
2.3458
2.3129
2.2067
2.1049
1.9892
1.6608
9.0 8.5 8.0 7.5 7.0 6.5 6.0 5.5 5.0 4.5 4.0 3.5 3.0 2.5 2.0 1.5 1.0 0.5 ppm
0.83
6.34
6.10
1.05
1.00
2.16
2.25
2.36
10.57
4.31
10.50
1.81

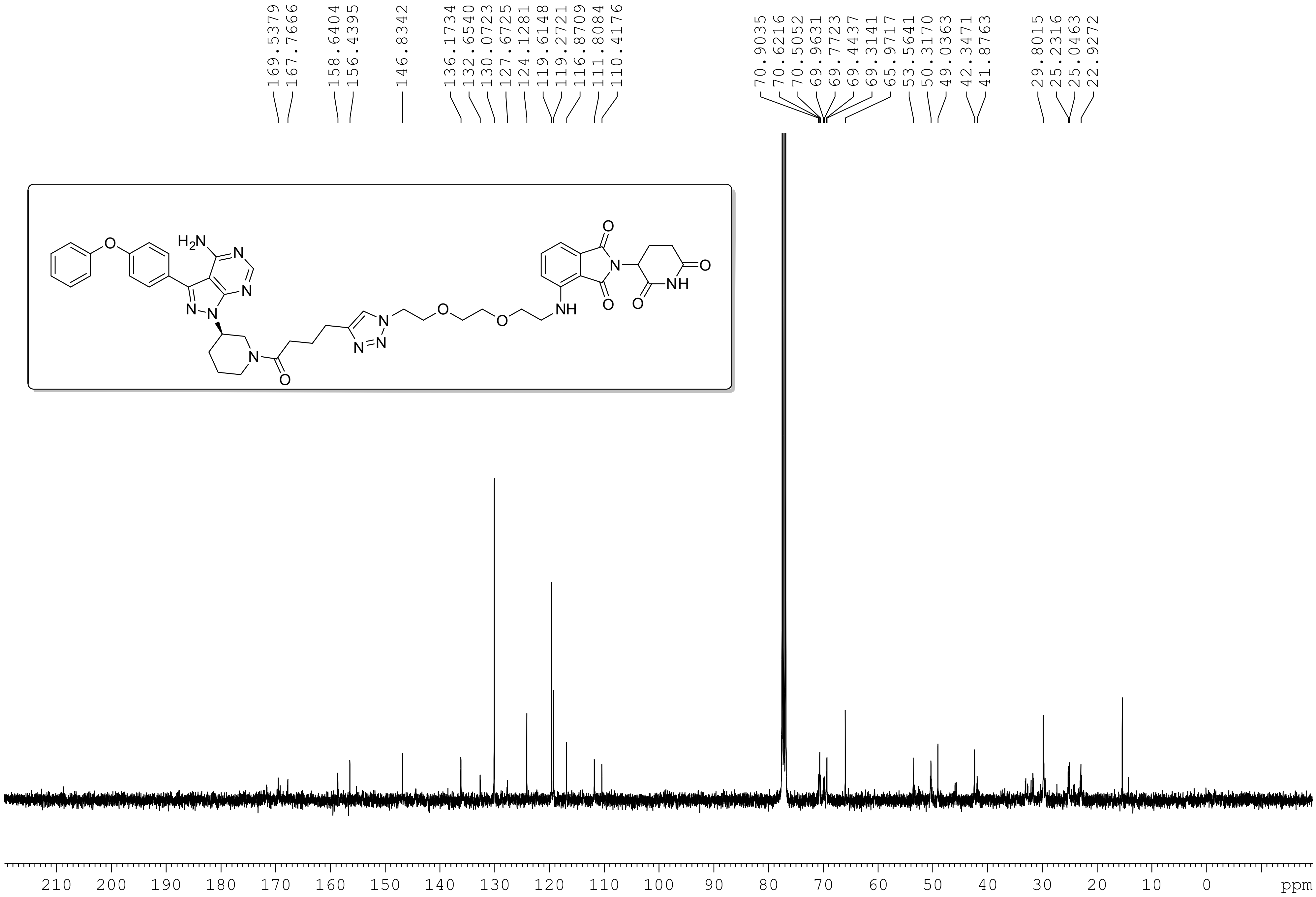
169.5379
167.7666
158.6404
156.4395
146.8342
136.1734
132.6540
130.0723
127.6725
124.1281
119.6148
119.2721
116.8709
111.8084
110.4176
70.9035
70.6216
70.5052
69.9631
69.7723
69.4437
69.3141
65.9717
53.5641
50.3170
49.0363
42.3471
41.8763
29.8015
25.2316
25.0463
22.9272
210 200 190 180 170 160 150 140 130 120 110 100 90 80 70 60 50 40 30 20 10 0 ppm
